# “Todo fue muy rápido y muy lento, a la vez”: experiencias heterocrónicas de profesionales de la salud en la prestación de ayuda a morir en España

**DOI:** 10.18294/sc.2026.5972

**Published:** 2026-05-20

**Authors:** Eduard Moreno-Gabriel, Maria Verdaguer, Patricia Beroiz-Groh, Xavier Busquet-Duran, Antonia Arreciado Marañón, Maria Feijoo-Cid, Miquel Domènech, Lupicinio Íñiguez-Rueda, Pere Toran-Monserrat

**Affiliations:** 1 Doctor en Psicología Social. Investigador, Unidad de Apoyo a la Investigación Metropolitana Norte, Fundación Instituto Universitario de Investigación en Atención Primaria de Salud Jordi Gol i Gurina, Mataró, España. emoreno@idiapjgol.info Fundación Instituto Universitario de Investigación en Atención Primaria de Salud Jordi Gol i Gurina Unidad de Apoyo a la Investigación Metropolitana Norte Fundación Instituto Universitario de Investigación en Atención Primaria de Salud Jordi Gol i Gurina Mataró Spain emoreno@idiapjgol.info; 2 Doctora en Psicología Social. Investigadora, Unidad de Apoyo a la Investigación Metropolitana Norte, Fundación Instituto Universitario de Investigación en Atención Primaria de Salud Jordi Goli i Gurina, Mataró, España. mverdaguer@idiapjgol.org Fundación Instituto Universitario de Investigación en Atención Primaria de Salud Jordi Goli i Gurina Unidad de Apoyo a la Investigación Metropolitana Norte Fundación Instituto Universitario de Investigación en Atención Primaria de Salud Jordi Goli i Gurina Mataró Spain mverdaguer@idiapjgol.org; 3 Doctora en Medicina. Médica, Hospital Germans Trias i Pujol, Badalona, España. pberoiz.germanstrias@gencat.cat Hospital Germans Trias i Pujol Hospital Germans Trias i Pujol Badalona Spain pberoiz.germanstrias@gencat.cat; 4 Doctor en Medicina. Investigador, Unidad de Apoyo a la Investigación Metropolitana Norte, Fundación Instituto Universitario de Investigación en Atención Primaria de Salud Jordi Gol i Gurina, Mataró, España. Profesor, Universidad de Vic- Universidad Central de Cataluña, Manresa, España. xbusquet@umanresa.cat Universidad de Vic- Universidad Central de Cataluña Universidad de Vic- Universidad Central de Cataluña Manresa Spain xbusquet@umanresa.cat; 5 Doctora en Ciencias de la Enfermería. Profesora, Departamento de Enfermería, Universitat Autònoma de Barcelona, Barcelona, España. antonia.arreciado@uab.cat Universitat Autònoma de Barcelona Departamento de Enfermería Universitat Autònoma de Barcelona Barcelona Spain antonia.arreciado@uab.cat; 6 Doctora en Antropología Médica. Profesora, Departamento de Enfermería, Universitat Autònoma de Barcelona, Barcelona, España. maria.feijoo@uab.cat Universitat Autónoma de Barcelona Departamento de Enfermería Universitat Autònoma de Barcelona Barcelona Spain maria.feijoo@uab.cat; 7 Doctor en Filosofía y Letras. Profesor, Departamento de Psicología Social, Universitat Autònoma de Barcelona, Barcelona, España. miquel.domenech@uab.es Universitat Autònoma de Barcelona Departamento de Psicología Social Universitat Autònoma de Barcelona Barcelona Spain miquel.domenech@uab.es; 8 Doctor en Filosofía y Letras. Catedrático, Departamento de Psicología Social, Universitat Autònoma de Barcelona, Barcelona, España. lupicinio.iniguez@uab.cat Universitat Autònoma de Barcelona Departamento de Psicología Social Universitat Autònoma de Barcelona Barcelona Spain lupicinio.iniguez@uab.cat; 9 Doctor en Medicina e Investigación Traslacional. Coordinador, Unidad de Apoyo a la Investigación Metropolitana Norte, Fundación Instituto Universitario de Investigación en Atención Primaria de Salud Jordi Gol i Gurina, Mataró, España. Profesor, Universitat de Girona, Girona, España. ptoran.bnm.ics@gencat.cat Fundación Instituto Universitario de Investigación en Atención Primaria de Salud Jordi Gol i Gurina Unidad de Apoyo a la Investigación Metropolitana Norte Fundación Instituto Universitario de Investigación en Atención Primaria de Salud Jordi Gol i Gurina Mataró Spain ptoran.bnm.ics@gencat.cat

**Keywords:** Eutanasia, Percepción del Tiempo, Práctica Profesional, España

## Abstract

Desde su entrada en vigor, la Ley Orgánica 3/2021 de Regulación de la Eutanasia ha modificado la experiencia y la práctica profesional de numerosas personas del ámbito sanitario en España. Este impacto se puede observar en la forma en que estos profesionales perciben las distintas temporalidades implicadas en el proceso. A partir de la noción de heterocronía, el presente artículo da cuenta de esta multitemporalidad mediante un análisis fenomenológico interpretativo de 29 entrevistas y tres grupos de discusión con profesionales implicadas en procesos de ayuda médica a morir. Las profesionales refieren una multiplicidad de tiempos superpuestos y en interacción: el tiempo de la ley, el tiempo del paciente, el tiempo familiar, los tiempos bio-farmacológicos, y el tiempo sociohistórico. Se destacan tensiones entre los plazos establecidos por la ley y los tiempos vividos por los solicitantes, o entre las temporalidades bio-farmacológicas y las lógicas profesionales vigentes. También se valoran los espacios temporales para acompañar a las familias y transitar hacia un paradigma profesional emergente. El manejo de esta heterocronía supone un reto para este nuevo rol. Sin embargo, el ensamblaje, sincronización y composición de los distintos tiempos podría facilitar una implementación armoniosa de la ley, mejorando la experiencia de todas las personas involucradas.

## Introducción

“Allá al fondo está la muerte, pero no tenga miedo. Sujete el reloj con una mano, tome con dos dedos la llave de la cuerda, remóntela suavemente. Ahora se abre otro plazo, los árboles despliegan sus hojas, las barcas corren regatas, el tiempo como un abanico se va llenando de sí mismo y de él brotan el aire, las brisas de la tierra, la sombra de una mujer, el perfume del pan. ¿Qué más quiere, qué más quiere? Átelo pronto a su muñeca, déjelo latir en libertad, imítelo anhelante. El miedo herrumbra las áncoras, cada cosa que pudo alcanzarse y fue olvidada va corroyendo las venas del reloj, gangrenando la fría sangre de sus rubíes. Y allá en el fondo está la muerte si no corremos y llegamos antes y comprendemos que ya no importa”. *Julio Cortázar*^1^

Con la aprobación en 2021 de la Ley Orgánica 3/2021 de Regulación de la Eutanasia (LORE)^2^, España se convirtió en el primer país mediterráneo en despenalizar la muerte asistida. Este nuevo marco legal implica un cambio social significativo, que afecta directamente a los profesionales sanitarios, quienes por ello han sido llamados a replantear su práctica asistencial^3^^,^^4^.

Para dar cuenta de las tensiones que experimentan las y los profesionales, es frecuente encontrar estudios sobre la eutanasia cuyos autores se apoyan de alguna manera en su etimología para comprenderlas^5^. De esta manera, dichos estudios sitúan los dilemas y conflictos profesionales en la relación entre el “buen morir” y el deber profesional, contribuyendo a la reflexión moral o disertando sobre la influencia de aspectos legales en las prácticas profesionales^6^.

Sin embargo, la entrada en vigor de la LORE y su implementación aporta una nueva dimensión a la eutanasia en España, en tanto que va dejando de ser meramente una entidad abstracta o una posibilidad más o menos tolerable, para devenir una práctica clínica concreta. En este sentido, para comprender qué significa la eutanasia para las y los profesionales en España, deviene necesario complementar los estudios que se interesan por cómo esta se construye a nivel discursivo, con enfoques praxiográficos que den cuenta de las prácticas que hacen realidad (enactan) la muerte asistida en España^7^. Desde esta perspectiva, los fenómenos y objetos biomédicos como la eutanasia dejan de concebirse como una entidad abstracta y estática ante la cual se puede estar a favor o en contra. Por el contrario, esta pasa a considerarse un proceso sociotécnico en el que se intentan coordinar múltiples ritmos y prácticas que, a su vez, son experimentadas en primera persona por las profesionales directamente implicadas en su implementación^8^. 

Tal y como mostraremos a continuación, practicar una eutanasia implica fundamentalmente gestionar y articular tiempos en tensión. Sin ir más lejos, la ayuda a morir puede considerarse una traducción al ámbito del final de vida de casos particulares de la orientación temporal hacia la anticipación que caracteriza las sociedades occidentales contemporáneas^9^. Concretamente, supone materializar el llamado “deseo de anticipar la muerte”, que emerge como posibilidad en estos contextos sociohistóricos para situaciones marcadas por un sufrimiento grave, crónico e imposibilitante^2^^,^^10^. Es decir, desde la perspectiva praxiográfica que tomamos como referencia, vida y muerte son constructos que emergen en determinados contextos discursivos. No obstante, se resalta cómo estos fenómenos son el resultado de prácticas sociomateriales que, como el hecho de dar cuerda al reloj, abren diversas temporalidades que, cuando se coordinan, permiten “correr y llegar antes” a la muerte, en palabras del escritor argentino Julio Cortázar. 

Así, en el presente trabajo nos disponemos de alguna manera a desplegar el abanico del tiempo y describir las distintas vivencias que, desde la perspectiva de las y los profesionales, convergen, se manejan y a veces colisionan en la implementación de la prestación de ayuda a morir desde la entrada en vigor de la LORE^2^.

### Marco contextual: el tiempo como problema práctico en la prestación de la ayuda a morir

**Figura 1:**
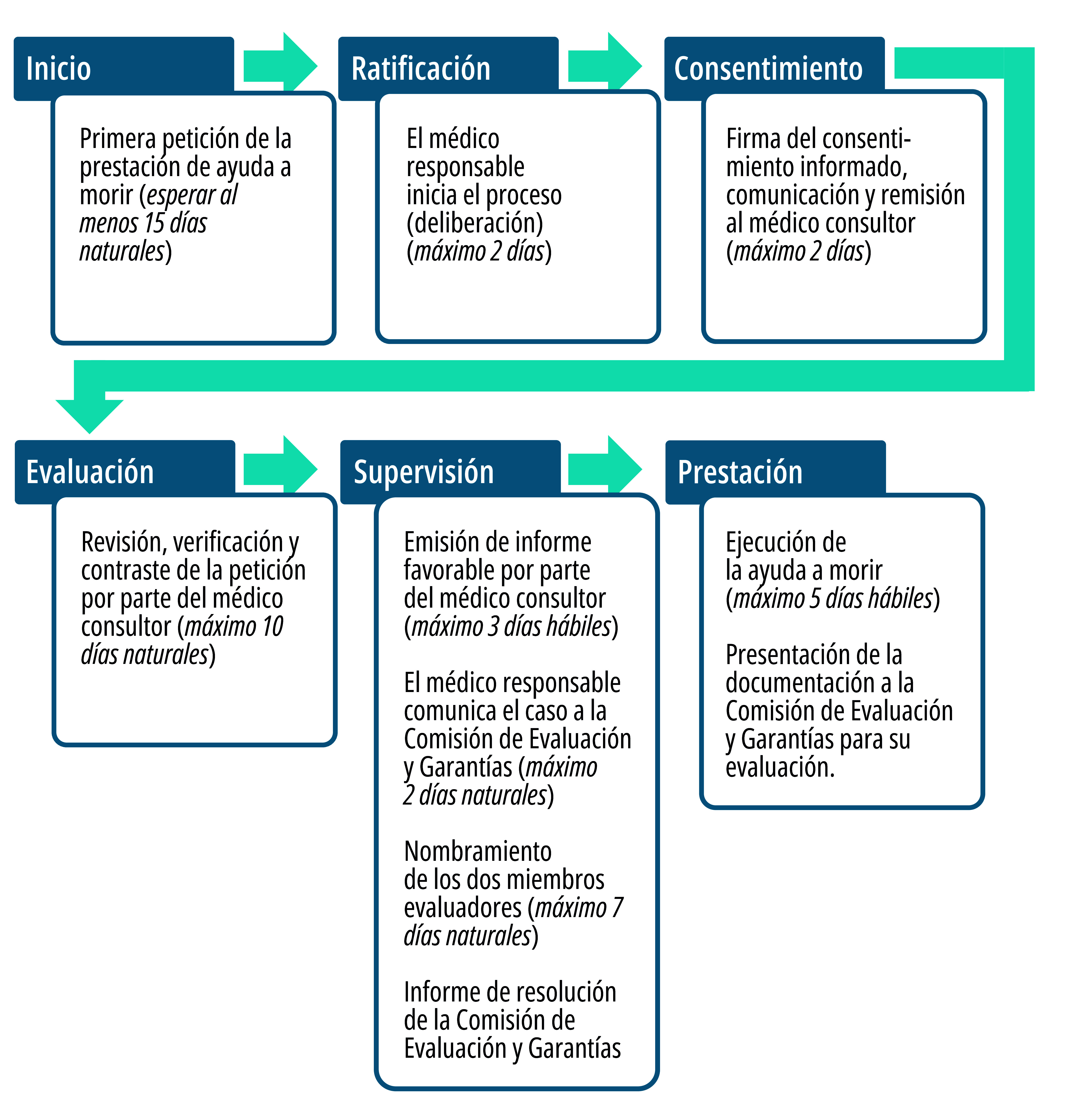
Proceso y tiempo desde la petición de la prestación de ayuda médica a morir hasta su realización en España, según la Ley Orgánica de Regulación de la Eutanasia.

A los pocos minutos de empezar a leer el texto de la ley, se puede constatar cómo la identificación, delimitación y estructuración de los tiempos se encuentra entre sus principales propósitos. Por un lado, para determinar los requisitos de acceso a este nuevo derecho sobresalen aspectos como la “cronicidad” del padecimiento o el hecho de tener un “pronóstico de vida limitado”. Por otro lado, con el objetivo de garantizar aspectos como la voluntariedad, la firmeza o el carácter deliberativo de la decisión, el derecho a reclamación, etc., el texto recurre constantemente al establecimiento de plazos temporales (por ejemplo, se impone la necesidad de formular dos peticiones con 15 días naturales de separación entre ambas). La Figura 1 muestra un resumen del proceso, incluyendo los tiempos establecidos.

Fuente: Elaboración propia sobre la base de Altisent *et al*.^11^

A pesar del esfuerzo legislativo por objetivar y concretar los tiempos del proceso de ayuda a morir, ya en el primer informe que elaboró el Ministerio de Sanidad español sobre el despliegue de la LORE, podemos observar cómo la gestión del tiempo y sus consecuencias emerge como uno de los principales problemas: “En algunos casos, el tiempo entre la segunda solicitud y el informe del médico consultor ha sido excesivo”^12^. Esta demora se sigue constatando en el último informe Catalán, en el que se identifica que un tercio de las personas solicitantes muere antes de recibir la prestación, provocando un aumento en la angustia generada en los solicitantes^13^.

Esta problematización de los tiempos se encuentra presente también en la literatura científica, aunque con algunas variaciones y matices^14^. En un artículo publicado en 2021, Altisent *et al*.^11^son críticos con la LORE precisamente con base en argumentos temporales. Reflejando un posicionamiento bastante extendido entre las y los profesionales de cuidados paliativos, estos autores se preguntan si la “ayuda a morir” sobre la que versa la LORE no podría limitarse al tratamiento sintomático en la agonía, sin que este suponga una aceleración de la muerte. Por otro lado, este trabajo también sugiere que las largas esperas para acceder a prestaciones sociales pueden estar influyendo en la decisión de solicitar la eutanasia. Finalmente, se identifican entre las cuestiones pendientes en la LORE tanto la formación como el tiempo de las y los profesionales para dar cabida a la necesaria deliberación.

Con relación a las diversas leyes australianas que regulan la eutanasia y la muerte asistida voluntaria, Wagner^15^ va un paso más allá de la crítica basada en el cumplimiento o el manejo de los tiempos para cuestionar la utilidad de tomar como referencia una visión lineal del tiempo para comprender las dinámicas de aceptación o rechazo de la ley y, por extensión, de la práctica eutanásica. Esta autora propone tomar como referencia la noción del péndulo perpetuo de Deleuze y Guattari^16^. Desde esta visión no lineal y fluida del tiempo, Wagner argumenta que es posible entender el carácter controvertido de este tipo de leyes, en tanto procesos dinámicos y en constante fluctuación entre el rechazo y la aceptación, lógicas que permiten ir más allá de la polarización para dar cabida a debates éticos y la constante discusión y evolución de las leyes.

Del breve recorrido entre textos legales, institucionales y académicos que rigen y reflexionan sobre la eutanasia, se desprende la importancia tanto de la estructuración como de la propia concepción de los tiempos para que la práctica de la ayuda médica a morir devenga posible^17^. Por esta razón, en el presente artículo nos proponemos explorar el tiempo desde la perspectiva de las y los profesionales implicadas en dicha práctica, previa articulación de una concepción propia del tiempo que permita observar su multiplicación y, a su vez, su coordinación en la singularidad de la prestación de la ayuda a morir^7^. 

### Marco conceptual: el tiempo como duración y heterocronía

Hemos podido constatar cómo la misma LORE y demás textos alrededor de la prestación de la ayuda a morir contienen y tratan de articular dos visiones del tiempo que son fundamentales para la comprensión de las dinámicas de salud, enfermedad (y muerte): el tiempo objetivo y el tiempo subjetivo. 

El psicólogo español Ramón Bayés, recientemente fallecido por eutanasia y una de las principales referencias teóricas en el abordaje de los aspectos emocionales en cuidados paliativos, recuperó esta dicotomía de la obra de William James para poner de manifiesto el impacto negativo de los tiempos de espera para las personas enfermas de cáncer y, en general de casi todo tipo de paciente. Concretamente, Bayés escribe:

“William James ya distinguía entre el tiempo objetivo y el tiempo subjetivo. Aun cuando el tiempo cronométrico ha sido imprescindible para elaborar la compleja organización del mundo actual -incluida la del mundo sanitario- y alcanzar extraordinarios avances tecnológicos, para el individuo, el tiempo que cuenta en su vida personal es el tiempo subjetivo y su duración es percibida como sumamente variable en función de su propia biografía personal, de sus expectativas y de los acontecimientos a los que, en cada momento, se halla expuesto”.^18^

Trasladado al caso que nos ocupa, si bien la LORE dedica amplio espacio a establecer tiempos objetivos para ensamblar el derecho a la ayuda a morir, para las personas implicadas en el proceso y parafraseando la célebre cita atribuida a Albert Einstein, *el tiempo que cuenta es precisamente el que no puede ser contado*. Especialmente si queremos comprender la eutanasia como práctica o proceso (asistencial, profesional, etc.) y no solo como construcción jurídico-legal, el tiempo objetivo deviene prácticamente irrelevante. 

Para prestar atención a esta vertiente más subjetiva y dinámica del tiempo, resulta pertinente situarse en contraposición a la visión más bien cuantitativa que Einstein tenía del tiempo. En el otro polo del debate, aunque con intención de complementar y no tanto refutar las teorías de Einstein sobre el tiempo, a principios del siglo XX se situó Henry Bergson^19^. Concretamente, Bergson propone el concepto de duración para comprender cómo el tiempo es vivido como algo dinámico y continuo, esencialmente indivisible si no es mediante una visión o un artefacto externo, como el reloj. A través de la idea de duración, Bergson se acerca más a dar cuenta del llamado “tiempo subjetivo”, en el que lo relevante es cómo se vive la sucesión y la mezcla o transición entre momentos que devienen indistinguibles^20^.

Así, cuando nos aproximamos al estudio del tiempo subjetivo, nos encontramos con una multiplicidad de duraciones que pueden superponerse, aunque la doctrina del tiempo objetivo no lo pueda concebir^21^. En el caso de la eutanasia como práctica asistencial, las y los profesionales registran, parcelan y describen sus tiempos a menudo mediante expresiones aparentemente contradictorias, como la que da título a este artículo. De esta manera, para comprender las vivencias y las prácticas profesionales alrededor de la prestación de la ayuda a morir, necesitamos comprender el tiempo como duración, tal cual es vivido y sentido, abarcando otras duraciones^22^. 

La teórica cultural y artista Mieke Bal^23^ se refiere a esta multiplicidad del tiempo como una característica especialmente distintiva del momento histórico actual en las sociedades occidentales. Esta autora aporta el concepto de heterocronía para definir esta experiencia de multitemporalidad:

“Así es como nos damos cuenta de que el tiempo no es homogéneo en nuestra experiencia. Debido a que con tanta frecuencia realizamos múltiples tareas, también estamos multitemporalizando: viviendo simultáneamente diferentes ritmos en el tiempo del reloj. El concepto de heterocronía ayuda a explicar las diferencias experienciales que conlleva enfrentarse, o estar sometido, al tiempo cronológico”.^23^

Bal introduce el concepto de heterocronía por primera vez en el marco de su exposición “*2MOVE: Migratory Aesthetics*”, un esfuerzo por concretar conceptos que, como el de heterocronía, faciliten un trabajo interdisciplinario en las humanidades como el que pretendemos en el presente trabajo^24^. Para esta autora, la migración es el estado que caracteriza nuestro mundo hoy en día, en el que todos estamos siempre en constante movimiento, entre la prisa, la espera y el estancamiento; así como navegando un presente inquietante que no se sustenta en un futuro predecible^24^.

Como el migrante, el profesional implicado en la prestación de la ayuda a morir se encuentra en una posición liminal^25^, en medio de una superposición de tiempos y ritmos que pueden ser paralizantes. Según Greco y Stenner^26^, esta encrucijada también puede señalar un cambio del paradigma en cuanto a la percepción generalizada del rol del profesional sanitario (especialmente, el médico), pasando de “salvador de vidas a tiempo completo” a “acompañante de procesos” capaz de articular la multi-temporalidad. Asimismo, en otro lugar, Stenner^27^ apunta que estos nuevo “yoes” profesionales emergen a su vez de la capacidad de situarse también entre distintas perspectivas, facilitando la armonización de los distintos tiempos que aporta cada una de ellas. George Mead nos recuerda que “debemos ser otros para ser nosotros mismos”^28^ y habitar lo que el autor denomina una socialidad liminal de transición, ya que es esta capacidad de asumir otros roles y tempos es lo que permite, a los profesionales, en este caso, participar y producir procesos colectivos como la eutanasia.

## Métodos

En el presente trabajo nos proponemos recoger esta diversidad de formas en las que se “cuenta” (en sentido narrativo ahora) cómo son experimentados los tiempos en la prestación de ayuda a morir en Catalunya (España). Concretamente, presentamos esta variedad desde la perspectiva de las y los profesionales implicados en el proceso de ayuda médica a morir.

Estas vivencias fueron recogidas en el marco de un estudio financiado por el Departamento de Salud del Gobierno Catalán, aprobado por el Comité de Ética en Investigación del Instituto de Investigación en Atención Primaria de Salud Jordi Gol (código 22/094-P), y llevado a cabo durante los primeros años de implementación de la LORE en Cataluña (2022-2024). Tal y como se describe en el protocolo y el artículo descriptivo publicados^29^^,^^30^, el grueso del estudio consistió en una serie de 21 entrevistas individuales de orientación fenomenológica a profesionales de la salud con implicación directa o indirecta en al menos un proceso de prestación de ayuda a morir en España. En total, las personas entrevistadas reportaron haber participado directamente en 45 procesos de ayuda a morir desde la entrada en vigor de la LORE. Concretamente, se entrevistó en profundidad a cinco enfermeros/as, diez médicos/as, tres trabajadoras sociales y tres profesionales de otras categorías. 

Las entrevistas tuvieron una duración media de una hora, y fueron llevadas a cabo de forma presencial, en distintas ubicaciones (mayoritariamente, en despachos cerrados, en el lugar de trabajo de la persona entrevistada), dado que se dio a escoger al participante el horario y el lugar que le permitiera exponer su vivencia de manera segura, confidencial y que facilitara su grabación. Todas las entrevistas fueron realizadas, tras la obtención del debido consentimiento informado, por una o un profesional de psicología, con el apoyo de un médico de familia. Dada la implicación directa del médico de familia como referente en el proceso de implementación de la LORE en el primer nivel de atención de la región metropolitana norte de Barcelona, el investigador principal del proyecto, ejerció de observador en las entrevistas, tomando notas que informarían los análisis posteriores. Sin embargo, su posicionamiento profesional facilitó el reclutamiento de participantes, a través de un procedimiento de bola de nieve, guiado por un muestreo teórico dirigido a entrevistar la mayor variedad de perfiles profesionales implicados en la implementación de la ley.

Con el objetivo de captar el mundo social y psicológico de las participantes, se optó por plantear entrevistas semiestructuradas en profundidad. Estas partieron de un guion elaborado a partir de una revisión bibliográfica y una ronda de ocho entrevistas exploratorias con informantes claves, con una visión panorámica de la ley en el contexto catalán, en su mayoría pertenecientes a la Comisión de Garantías y Evaluación de dicha comunidad autónoma. Sin embargo, dicho guion no fue prescriptivo, y las conversaciones se orientaron a facilitar al máximo que las personas pudieran contar su historia, siguiendo así los preceptos del método fenomenológico interpretativo^31^. 

Finalmente, en tanto que el estudio mayor financiado preveía la elaboración de una guía de buenas prácticas, las entrevistas se complementaron con seis grupos de discusión distribuidos en dos fases. En la primera fase, llevada a cabo de forma concurrente a las entrevistas fenomenológicas, se buscó especialmente la triangulación metodológica, mientras que en la segunda fase (llevada a cabo tras las entrevistas fenomenológicas) se priorizó la cocreación de propuestas de mejora y buenas prácticas en la implementación de la LORE. Todas las entrevistas y grupos de discusión se grabaron en audio, y se transcribieron literalmente por parte del equipo investigador y una persona externa, preservando la confidencialidad de los datos en todo momento.

Como resultado del análisis temático conjunto del set de datos, además del análisis presentado en este artículo, ya se han publicado dos artículos. Por un lado, en Verdaguer *et al*.^30^, hemos puesto de manifiesto las fuentes de malestar identificadas en los relatos de las participantes, como bases para la elaboración de una guía de buenas prácticas que está en proceso de construcción. Por otro lado, en Feijoo-Cid *et al*.^32^, hemos considerado necesario visibilizar el papel de las profesionales de enfermería durante todo el proceso, ya que es un perfil poco estudiado y reconocido tanto en la literatura académica como en los documentos legales y oficiales.

En el presente artículo presentamos los resultados de las 8 entrevistas exploratorias, las 21 entrevistas fenomenológicas y los 3 grupos de discusión realizados en la primera fase del estudio, analizados siguiendo los procedimientos del análisis fenomenológico interpretativo^33^^,^^34^con el objetivo de indagar en profundidad y comprender las vivencias profesionales. El análisis se llevó a cabo de manera circular, prestando atención a los temas emergentes de los datos y, a su vez, contrastando esos constructos emergentes con el conjunto de los datos. Concretamente, se partió de un abordaje idiosincrático de las entrevistas para, posteriormente, llevar a cabo una integración de los casos. De esta manera, las primeras impresiones -tanto al escuchar las entrevistas como al leer sus transcripciones una a una- hicieron emerger la centralidad del tiempo vivido como una de las principales categorías (o clústers) alrededor de la cual las personas entrevistadas organizaban sus relatos. Por lo tanto, se tomó esta categoría principal y sus distintas manifestaciones para estructurar los temas presentados en los resultados (es decir, los distintos tiempos), como referentes contra los que contrastar y re-codificar el conjunto del corpus. En este trabajo analítico participaron cuatro de los autores firmantes del artículo, facilitando así la triangulación de los datos y garantizando la rigurosidad del análisis.

## Resultados

Como suele ocurrir en las investigaciones cualitativas, ya en las entrevistas se empezaron a elaborar las primeras intuiciones analíticas. Para describir sus experiencias, las participantes se apoyaban a menudo en referencias directas o indirectas tanto a su percepción del paso del tiempo (o duración) como al impacto que les producían otros tiempos o ritmos que percibían más bien como externos. Más allá de las descripciones detalladas de sus vivencias subjetivas, cabe destacar cómo se explicitaba la con-vivencia de estas distintas temporalidades en un solo momento:

“*Todo fue muy rápido y muy lento a la vez* [...] C*laro, si tú te pones a mirar desde que llegamos al hospital hasta que estas dos personas murieron, creo que pasaron dos horas. Pero, en el momento de poner las inyecciones, sí fue muy rápido. Además, nosotros tampoco sabíamos... Yo sí tenía un poco de conocimiento del efecto que tienen estas drogas, pero no estas dosis. Claro, son dosis extraordinarias. Pero claro, lo que estás buscando precisamente es que estas personas se mueran y, además, rápido. No es una sedación, no es un tiempo... No. Es ya. Es el momento que lo pones hasta que... Creo que pasaron diez minutos. Tampoco lo contamos, pero diría que pasaron diez minutos desde que lo pusimos... Pero sí que es verdad que la sensación que yo tenía, sobre todo cuando preparaba la medicación hasta que llegó el momento de entrar a la habitación y poner la medicación, es como una... distorsión del tiempo, ¿no?* (EF:118, enfermera de atención primaria)

**Figura 2:**
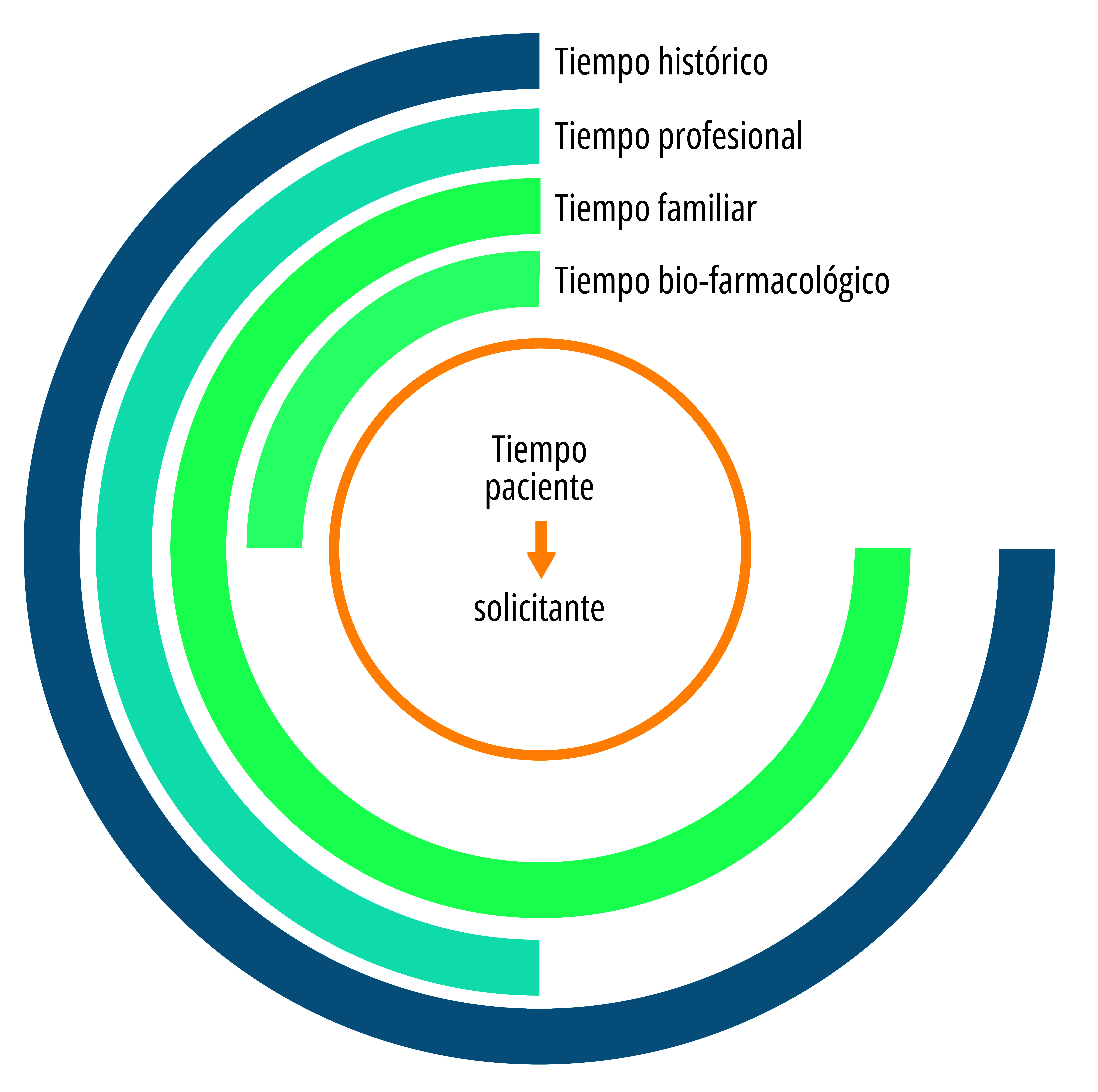
Temporalidades en la implementación de la ayuda médica a morir.

Por esta razón, a continuación, recogemos las distintas temporalidades que fueron emergiendo en nuestras entrevistas, tratando de mantener también visible esta superposición. Para ello, invitamos al lector a imaginar el tiempo como una espiral (Figura 2). En el centro, situamos el tiempo de la paciente que transita hacia la muerte, pasando por su posición de solicitante. Este tiempo se encuentra incrustado y atravesado por otros ritmos y círculos temporales que van desde el devenir histórico que ha permitido la emergencia de la LORE, hasta los tiempos que los fármacos utilizados en la prestación necesitan para actuar^35^.

Fuente: Elaboración propia.

Consideramos pertinente iniciar la exposición de temporalidades por la vivencia profesional, en tanto que es la experiencia narrada en primera persona por las participantes en el estudio. A continuación, seguimos los vínculos que este colectivo va trazando en su discurso, vinculándose a otras vivencias y velocidades, por este orden: paciente-solicitante, familia, bio-farmacología e historicidad. 

### Tiempo de profesional: tiempo intangible e insólito

Más allá del ámbito de final de vida, es habitual que el tiempo surja como un elemento clave para describir las vivencias de las y los profesionales de la salud. Frecuentemente, en contextos de elevada presión asistencial, como el primer nivel de atención en España, el tiempo profesional es percibido como un recurso escaso. Este da lugar a ritmos cada vez más acelerados, con los consiguientes relatos de malestar por parte de sus profesionales. A menudo, y también en nuestros datos, este elemento temporal surge para articular una demanda cuantitativa de disponer de más tiempo:

“*Y luego hay un tema que para mí es esencial, que es el tiempo. El tiempo que el médico responsable y el consultor también tiene que dedicar cuando llega una demanda de eutanasia, es un tiempo que no tienen muchas veces y esto les estresa mucho.* […] *La insistencia en apretarlos con eso es más para tener las cosas bien formalizadas, pero es verdad que, como sistema, como modelo asistencial, deberíamos hacerlo de modo que cuando un profesional recibe una de estas demandas tenga el tiempo para aplicarla. Y, por lo tanto, los días que necesite para cerrar este expediente se pueda liberar la agenda, porque realmente, esto, el estrés psicológico que le puede suponer... Requiere tiempo para hacerlo. Yo creo que a veces hay denegaciones que son por falta de tiempo, porque dicen: ‘Fuera’*”. (EE03:88, jurista, mujer, miembro de la Comisión de Garantía y Evaluación)

Incluso, tal y como vemos en el discurso de esta médica, la falta de una cantidad de tiempo suficiente se apunta como una posible explicación de los rechazos explícitos o implícitos, por parte de profesionales no objetores, a participar en la implementación de la prestación de ayuda para morir. A su vez, estos rechazos/retrasos generan una dilatación temporal en todo el proceso. Sin embargo, nuestras participantes van más allá de una valoración cuantitativa del tiempo necesario para llevar a cabo la prestación, para poner de relieve la intensidad de la tarea a realizar y cómo esta desborda el tiempo previsto para la prestación, permeando tanto en el pasado como en los momentos posteriores:

“*…el primero, que es* [nombre del solicitante], *yo estuve 15 días sin dormir. Porque yo pensaba: ‘Es que no se me puede escapar nada’*”. (EE01, médica de hospital)

“*Y luego, sobre todo, tiempo. Otra cosa muy grave que creo que está pasando es que... Sabéis lo que está pasando a nivel de que hay mucha carga asistencial... Entonces, es muy difícil dedicar el tiempo necesario para digerir, para pensar, para acompañar qué haría falta cuando tienes tanta presión asistencial. A veces, compañeros han tenido que hacer una PRAM* [prestación de ayuda para morir] *y luego ir a un domicilio. Esto es mucho. Necesitas parar para metabolizar este acompañamiento, esta imagen bonita o emotiva que has percibido, necesitas un rato para digerirlo. Y a veces no tienen tiempo y más si vamos a* [atención] *primaria. Yo lo sé porque tengo muchas amigas y amigos que están en primaria y que tienen esta dificultad de mucha presión asistencial, un ritmo muy loco y el deseo de acompañar y dar respuesta a las necesidades de los pacientes desde un lugar muy auténtico. Pero me pregunto si tienen suficiente espacio. A lo mejor una forma de cuidar es que una persona que practique una PRAM solo pueda hacer esto ese día. A lo mejor es dar el tiempo suficiente para que luego puedas ir a la playa a dar un paseo o tomarte un té. Quién dice un té dice un vinito... pero parar. Tengo la sensación de que eso, a veces, no puede pasar*”. (EE05:70, psicóloga)

Aunque se aprecian similitudes con otras prácticas clínicas, nuestras participantes caracterizan distintivamente una prestación de ayuda para morir como algo que implica una preparación, un acompañamiento, una metabolización (en palabras de la participante que citábamos anteriormente) e incluso una parada específica. Desde la perspectiva de las profesionales entrevistadas, implementar la eutanasia requiere unos tiempos cualitativamente y cuantitativamente diferenciados y, a su vez, difíciles de concebir en la organización actual de la práctica asistencial en España. Además de “intangibles”, estos tiempos se consideran insólitos, fuera de la lógica y la planificación sanitaria:

“*Claro, yo he participado en 24* [eutanasias] *de alguna manera. Eso son muchas horas, muchas horas, muchos fines de semana, festivos, traspasar las vacaciones porque hay un procedimiento abierto.* […] *Pero claro, mis compañeros tienen necesidades, y tienen necesidades a veces insólitas. ¿Y qué pasa? Te conectas, hablas, claro... Todo esto es intangible, pero está. Y, claro, es que a mí me supone mucho trabajo*”. (EE01:10, médica de hospital)

En este caso, esta profesional también nos habla de alguna manera de los efectos y la sobrecarga que conllevan las objeciones que hemos mencionado más arriba. Por ahora, en Catalunya, las y los profesionales que conocen bien los tiempos y los procesos que conlleva un proceso eutanásico no son muchos. Por esta razón, para el devenir del proceso se torna inevitable que el tiempo y la disponibilidad de estas personas, que actúan como referentes para resolver dudas durante el proceso, se estire y se sature de formas tan extremas como inapreciables.

### Tiempo del (im)paciente: hacia la des-esperación del solicitante

Tal y como hemos visto en los extractos referentes a la perspectiva profesional, entre las demandas que formulan las y los profesionales con relación no solo a la cantidad, sino a la calidad de los tiempos necesarios e implicados en la prestación, emerge la importancia de un proceso indefinido y relacional, como es el del acompañamiento y la creación del vínculo de confianza médico-paciente:

P1: “*Además, hay una cosa que es que el vínculo… va ligado a la confianza. La confianza no es cuestión de que el paciente tiene confianza en el médico, es que el médico tiene confianza en el paciente. La confianza es mutua, esto es muy importante*”.

P2: “*Y se crea con el tiempo. No llegas a una consulta y ya allí hay confianza*”.

P1: “*Y eso facilita mucho las cosas*”. (GD02:157, profesionales de la salud participantes, en grupo de discusión)

De esta manera, vemos cómo la perspectiva de la persona solicitante entra en la duración de la persona profesional que asiste, y requiere de una reciprocidad para que el proceso sea más fácil. Es decir, si bien es difícil cuantificar el tiempo necesario para establecer la confianza (ya que depende de la interacción y la coordinación de al menos dos perspectivas), se trata de un tiempo y de una perspectiva que son reconocidas por las y los profesionales.

Desde su posición profesional, el tiempo de las personas solicitantes se podría dividir al menos en dos momentos o, mejor dicho, dos posiciones (porque se solapan y pueden simultanearse): el tiempo como paciente y el tiempo como solicitante. En tanto que nuestra investigación se centró en explorar las vivencias durante el proceso de implementación de la LORE, en nuestros datos tenemos pocas referencias al tiempo previo a la decisión de la solicitud. Sin embargo, podemos entrever que en muchos casos se hace referencia a un proceso más o menos progresivo, en el que una persona va transitando desde una posición de “paciente” (en un doble sentido: insertada en un sistema sanitario que la trata como tal y que, al mismo tiempo, es capaz de saber esperar y aceptar la lentitud del devenir de la enfermedad) hacia una subjetividad más “activa”, que toma las riendas y decide solicitar la eutanasia, sobreponiéndose (en el sentido de acumular, no tanto de superar) la posición de “paciente” y deviniendo alguien más bien “impaciente”, que ya no puede esperar.

“*Para los pacientes, cuando ya lo tienen decidido, este tiempo de espera se les hace largo, cuando lo tienen como todo muy cuadrado*”. (EF02:739, enfermera de atención primaria)

“*Yo recuerdo, en las primeras solicitudes, cuando se hacían las segundas solicitudes, en el apartado de observaciones, la queja del solicitante -había muchas quejas- y era: ‘¿Hasta cuando tengo que esperar? Ya lo he decidido. ¿Por qué me hace esperar tanto?’. Porque cuando ya has dado el paso, supongo que al que pide la prestación se le debe de hacer eterno*”. (GD01:61, jurista, hombre, miembro de la Comisión de Garantía y Evaluación, en grupo de discusión)

Tal y como muestran las observaciones a las que hace referencia esta profesional, la determinación e incluso la prisa de la persona solicitante choca a menudo con los tiempos establecidos por la ley. Sin embargo, a continuación, veremos otra perspectiva desde la que estos plazos toman un nuevo sentido.

### Tiempo de la familia: entre la comprensión y la esperanza

“*Yo pienso que a las familias las ayuda a poder entender, porque hay familias que lo entenderán y familias que no. Pero yo pienso que este tiempo sí que los ayuda a poder entender la decisión de su familiar. Ya sé que para el familiar* [solicitante] *es una putada, si yo os digo que ya tengo bastante, ¿todavía me hacéis esperar un mes más? Ostia. Por el paciente lo entiendo, pero a la familia sí que es verdad que este tiempo pienso que les ayuda*”. (EF01:66, enfermera de cuidados paliativos hospitalarios.)

De nuevo, en este extracto, observamos un contraste y una yuxtaposición de miradas, afectos y efectos vinculados a un mismo tiempo. Concretamente, este profesional nos habla del plazo de 15 días provisto en la ley para que la persona solicitante corrobore su decisión. Este tiempo puede ser visto no solo como innecesario, sino incluso como un agravio (una “*putada*”) desde la perspectiva de la persona solicitante. Sin embargo, según las profesionales, es un tiempo que se vive de forma distinta desde la perspectiva de sus personas allegadas, en tanto que abre espacios para entender la decisión. 

Y en este juego de perspectivas temporales que interactúan (la ley, el paciente, la familia…), también se dan sincronizaciones que pueden dejar en segundo plano a la persona solicitante, quien, *a priori*, debería conducir el proceso. Por ejemplo, estos mismos tiempos legales permiten también la reflexión profesional (quizás para compensar la escasez temporal referida más arriba) y, de manera más o menos consciente, hacen emerger esperanzas compartidas por algunos profesionales y algunos familiares:

“[El período de] *reflexión no es por el paciente, porque ellos ya lo tienen clarísimo y ya vienen más que reflexionados. De hecho, yo decía, esto lo tengo que cambiar, es por el profesional. Para que haga este viaje de si realmente puede hacer este trabajo y si lo puede hacer hasta el final*”. (EF01:91, enfermera de cuidados paliativos hospitalarios)

“*Cuando supimos la fecha…* […] *Vas a hablar con el paciente y siempre dices: ‘Se echará atrás…’ pues no. Dejemos pasar el tiempo… y no.* [...] *Y la familia también esperando que en último momento dijera que no. La familia esperando*”. (EF22:30, enfermera de atención primaria)

### Tiempo bio-farmacológico: “yo no tengo 45 días”, “yo tengo reserva, arsenal terapéutico”

Más allá de las vivencias personales, familiares y profesionales, los relatos de las participantes nos invitan a adoptar una perspectiva simétrica del proceso eutanásico, y entenderlo como una práctica sociotécnica^36^. Concretamente, las participantes nos muestran cómo en esta praxis se articulan los tiempos vividos por las personas implicadas con la intervención de las temporalidades que podríamos llamar “no-humanas”, para facilitar la mirada analítica.

En primer lugar, la perspectiva de las personas solicitantes que hemos visto recogida en la mirada del personal sanitario a menudo incorpora las posibilidades y los tiempos marcados por la fisiología de sus cuerpos (“*yo no tengo 45 días*”).

“*No hace falta que te esperes 45 días a que ‘tropecientos’ comités firmen papeles. Este también es un tema que genera mucho malestar. ‘¿Por qué me tengo que esperar yo 45 días? Me parece injusto, no hay derecho, yo no tengo 45 días...’ añadimos una angustia porque no saben si llegarán al final del trámite*”. (EF17:36, enfermera de atención primaria)

“*Hasta finales de octubre no conseguimos hacer esta primera solicitud oficial... Finalmente, no llegamos a... Hicimos la segunda solicitud, pero fue un éxitus prematuro porque empezó con una infección multisistémica y lo derivamos a domicilio y lo sedé*”. (GD03:40, médico de atención primaria, en grupo de discusión)

“*Todo estaba bien, pero por los timings no llegamos. El señor, en un fin de semana, encima no era ni entre semana, yo tampoco estaba y, con personal que no conocíamos tanto, lo tuvieron que asistir, un sábado para domingo, de madrugada. El señor con una hemorragia digestiva alta y una situación crítica a domicilio*”. (GD01:51, médico de atención primaria, en grupo de discusión)

Tal y como se intuye en esta última cita, los tiempos del cuerpo-enfermedad y sus posibilidades también entran en contacto y en conflicto con otras dinámicas temporales. En este caso, vemos cómo la organización social del tiempo laboral no siempre logra sincronizarse con los “*timings*” de, por ejemplo, un aparato digestivo ya muy envejecido o precario. La fragilidad clínica en ocasiones genera una descompensación que requiere una atención urgente, en fin de semana y sin la mediación de la confianza cultivada durante meses por el o la profesional de referencia.

Por otro lado, algunos cuerpos aguantan hasta la fecha establecida para la prestación, y quién sabe si más allá. Tal y como vemos en el siguiente extracto, la mirada más puramente biomédica recurre a elementos materiales (como el “arsenal terapéutico”) para evidenciar un desacuerdo con la voluntad de la persona, precisamente alrededor de la cantidad de tiempo que debe ser vivido por esa persona (“*todavía tiene opciones*”):

“*Al revés, la ley es clarificadora. Es la voluntad de la persona y el contexto eutanásico. Es mucho más complicado entorno los críticos* [pacientes en unidades de cuidados intensivos]*, por ejemplo, los temas de limitación. Aquí sí que patinamos que da gusto. ‘Porque yo no lo veo claro’, ‘yo pienso que todavía tiene opciones’, ‘yo tengo reserva, arsenal terapéutico para ir haciendo’*”. (GD02:214, enfermera de atención primaria, en grupo de discusión)

En aquellos casos en que llega el día de la prestación, la farmacología toma un mayor protagonismo si cabe, normalmente por su velocidad. En considerables ocasiones, el recuerdo que perdura durante un mayor tiempo, volviendo a la memoria en momentos inesperados, es un instante marcado por una especie de irrealidad temporal, ya sea por la aceleración o la lentitud del tiempo en que tarda en actuar el fármaco.

“*Me afectó más al terminar. Yo me sentía tranquila, pero, por la noche me vino la imagen de él, del cambio de color, es que es inmediato. Eso sí que fue un impacto, qué deprisa llega la muerte*”. (GD01:39, enfermera de atención primaria, en grupo de discusión)

“*A partir de aquí, es este momento que he dicho que el tiempo se borra, no hay medida del tiempo. Intentas hacerlo fácil dentro de lo que tú crees que tienes que ir haciendo con la inyección, la administración de fármacos, pero pierdes la realidad, es un momento en que pierdes la realidad. Y si, encima, hay alguna traba, que es mínimo, como que tú pones y piensas ‘ostras, todavía se está despidiendo’, claro, entras en pánico.* […] *Después fue muy fácil, fue muy fácil. Fácil, pero tú tienes ganas, como médico, de que esto se acabe rápido, porque tú estás sufriendo. Te pones el fonendo y aquello late, el corazón sigue latiendo. ‘Ay, Dios, ¿que he hecho mal?’ Ya te estás planteando que tienes que abrir el segundo ‘kit’ porque con uno no hemos tenido bastante. Tú crees que ya han pasado muchos minutos y vuelves a poner el fonendo, continúa latiendo. Entras en pánico, de verdad, por mí ha sido un momento tan complicado. Y el referente dice, no, vamos bien, tenemos que esperar, vamos con el reloj, pero por el que eran dos minutos, yo pensaba que habían pasado 10 como mínimo. Y seguía latiendo. Porque lo que quieres es acabar, quieres marchar, quieres marchar. Ya has hecho todo lo que tenías que hacer y tienes el corazón encogido y quieres marchar, como sea*”. (EF08:28, médico de atención primaria y psiquiatra)

Este extracto resulta especialmente revelador de la distorsión temporal (“*el tiempo se borra*”) que acompaña todo el proceso y cómo esa distorsión se vuelve especialmente manifiesta en el momento de la prestación. A su vez, podemos observar cómo esta persona se sitúa en medio de distintos tiempos, tratando de darles una cierta coherencia: el profesional, “como médico”, que le pide premura; el técnico-farmacológico, que aporta una cierta expectativa de estructuración que facilite el proceso, pero se ve modelado por un corazón que sigue su tempo, su latido, más allá del tiempo objetivo marcado por el reloj, etc.

### Tiempo sociohistórico

La tensión que emana del vívido relato citado anteriormente se explica en parte por la colisión de dos temporalidades que dan lugar a la percepción de rapidez fruto del efecto de la medicación. Esta sensación de aceleración emerge de la comparativa entre la acción de los fármacos (o sus dosis) utilizados en la eutanasia, con la duración de los procedimientos que históricamente, hasta la entrada en vigor de la ley, eran los más habituales para los profesionales, como la sedación paliativa.

“*Has visto alguien morir así, en nada. Porque, claro, yo aquí veo muchos procesos de sedación, pero claro, es muy progresivo, no es esto tan rápido. Y desde este nivel de conciencia de, ya estoy preparado, ya podemos empezar. Me impactó esto, pero yo al día siguiente ya estaba*”. (EF06:63, profesional hospitalaria de atención espiritual)

Es decir, el impacto de los tiempos dictados por las medicaciones propias del procedimiento eutanásico surge en un contexto determinado, que cultiva un “nivel de conciencia” particular y preparado para acompasar ciertos ritmos. En cambio, el poco tiempo transcurrido desde la entrada en vigor de la LORE hasta el momento en el que se realizaron estas entrevistas (entre dos y tres años) no ha permitido a las y los profesionales responsables de administrar dichas medicaciones recibir formación y aclimatarse a estas nuevas temporalidades farmacológicas. De hecho, uno de los mayores efectos de esta nueva ley tiene que ver con un cambio en las subjetividades profesionales, que se encuentran transitando hacia un nuevo escenario:

“*La gente confunde los tratamientos, por ejemplo, en este caso, otra cosa también que pasó es que el equipo se encontró que no tenían ninguna duda de lo que tenían que hacer, de cómo lo tenían qué hacer, pero claro, no lo habían hecho nunca. Era una medicación absolutamente nueva que nadie te ha hecho una… no habían tenido ninguna formación sobre cómo utilizarla, qué cosas podían pasar.* [...] *Entonces, no es lo mismo poner una sedación paliativa, que sabes que estás poniendo una Buscapina®, un fentanilo o un midazolam, que sabes cuál es el efecto que hará, que poner un propofol o un (ininteligible) que no tiene nada que ver.* [...] *Los médicos no habían puesto nunca una medicación que promovía la muerte*”. (EE05:36, psicóloga)

Asimismo, este “tiempo de maduración social” (parafraseando a la participante que recogemos a continuación), que de momento se considera insuficiente para la emergencia de profesionales formados, sí ha permitido que se apruebe una ley determinada. Tanto los tiempos (44 días) como la secuencialidad (autorización “pre” vs. control “post”) específicas que esta ley propone, son aquellas que sus redactores consideran tolerables en el contexto sociohistórico y sociotécnico, como atestigua la referencia a la plataforma informática de Prestación de Ayuda para Morir (PRAM) en la siguiente cita, en que se promulgan. Finalmente, esta misma cita que recogemos aquí reafirma esta dimensión sociohistórica de la práctica eutanásica al traer a colación lo que ocurre en otras latitudes (Holanda, Bélgica, etc.), abriendo la posibilidad de que, en el contexto español, las cosas pueden distintas en el futuro.

“*El proceso en sí es doloroso.* [...] E*s doloroso también porque la ley en su aplicación es dolorosa. El proceso, como mínimo de 44 días si las cosas van bien. Después hay quien dice también que tenemos una ley que es autorización… Antes, las leyes holandesas y belgas son otras leyes, es decir, se controla a posteriori, pues esto genera un sufrimiento, además de por toda la burocracia y aquí en Cataluña esto pasa mucho, porque el programa PRAM que hicieron desde la consejería en su momento es un desastre.* […] *Además del procedimiento en sí, que es pesado, es largo, pues claro, esto lo hace más difícil. Y después lo que decíamos de la progresión de antes, esto dicen que genera más sufrimiento porque la vinculación médico-paciente se ve asaltada e invadida por un proceso administrativo que no tendría que existir. Lo cierto es que la confianza de la sociedad posibilitó que tuviéramos esta ley, es decir, yo soy partidario de que la progresión fuera de otro modo, pero seguramente la madurez de la sociedad no permitía… Las leyes son siempre aproximaciones y una primera aproximación que se ha hecho aquí es esta con todas las limitaciones que tiene, pero no ha sido posible hacer otra. Esto también genera sufrimiento: cómo es la ley. La burocracia, el tiempo, la aprobación posterior, la aprobación anterior, pasar por una comisión…*”. (EE04:16, psicólogo bioeticista, miembro de la Comisión de Garantía y Evaluación)

En definitiva, del relato de las personas participantes destacan no solo las distintas temporalidades que atraviesan el proceso, sino también la diferencia en la magnitud de sus escalas, yendo desde el ritmo de un corazón hasta el ritmo de maduración social, pasando por los plazos dictados por la ley. Sin embargo, por encima de la multiplicidad de tiempos, destaca cómo se perciben las interacciones entre ellas. En este sentido, sobresale especialmente en la mayoría de las entrevistas la percepción de que los tiempos burocráticos “asaltan e invaden” (en palabras de la última cita) el proceso de elaboración de un vínculo social, como es la relación médico-paciente, una relación formada por subjetividades que se encuentran en transición. 

## Discusión

“Piensa en esto: cuando te regalan un reloj te regalan un pequeño infierno florido, una cadena de rosas, un calabozo de aire. No te dan solamente el reloj, que los cumplas muy felices y esperamos que te dure porque es de buena marca, suizo con áncora de rubíes; no te regalan solamente ese menudo picapedrero que te atarás a la muñeca y pasearás contigo. Te regalan -no lo saben, lo terrible es que no lo saben-, te regalan un nuevo pedazo frágil y precario de ti mismo, algo que es tuyo pero no es tu cuerpo, que hay que atar a tu cuerpo con su correa como un bracito desesperado colgándose de tu muñeca. Te regalan la necesidad de darle cuerda todos los días, la obligación de darle cuerda para que siga siendo un reloj; te regalan la obsesión de atender a la hora exacta en las vitrinas de las joyerías, en el anuncio por la radio, en el servicio telefónico. Te regalan el miedo de perderlo, de que te lo roben, de que se te caiga al suelo y se rompa. Te regalan su marca, y la seguridad de que es una marca mejor que las otras, te regalan la tendencia a comparar tu reloj con los demás relojes. No te regalan un reloj, tú eres el regalado, a ti te ofrecen para el cumpleaños del reloj”.^1^

En el presente trabajo hemos recogido las visiones de las y los profesionales implicados en la implementación de la Ley Orgánica de Regulación de la Eutanasia en España, constatando la relevancia que le otorgan a la percepción del tiempo para describir sus vivencias. Entre otros, las personas participantes recogen y describen los tiempos burocráticos, profesionales, familiares, farmacológicos, sociales y, evidentemente, el tiempo del paciente en su devenir solicitante. 

Con relación a este último, las y los profesionales cuentan cómo las personas solicitantes catalogaban el hecho de poder anticipar su muerte como “*el mejor regalo que le podríais haber dado*” (EF22:30, enfermera de atención primaria). Sin embargo, tal y como nos advierte Cortázar en su preámbulo, algunos regalos, y concretamente un reloj, puede ser un “infierno florido” en tanto que teje nuevas relaciones socio-materiales que conllevan una serie de obligaciones y necesidades emergentes. En consonancia, diversos estudios sobre las experiencias profesionales en la implementación de la prestación de la ayuda a morir, caracterizan estas vivencias con una fuerte ambivalencia y emociones contradictorias^37^^,^^38^.

A lo largo de las entrevistas realizadas a profesionales en el marco de nuestro proyecto, hemos podido captar algunas de estas tensiones introducidas, en este caso, por la ley y sus tiempos, así como los malestares que conllevan^30^. Sin embargo, en ocasiones, estos profesionales también reciben los agradecimientos de sus pacientes por este “*regalo*”. De hecho, la implementación de la ley permite a muchas personas transitar de la posición de pacientes a solicitantes de eutanasia, generando una nueva espera, pero también empoderamiento^39^. En este sentido, Pesut *et al*.^40^explican esa sensación de gratitud de las personas solicitantes: establecer un plazo y una fecha para el morir significa, en cierto modo, retomar el control sobre el tiempo que les queda y, por extensión, sobre su propia vida.

En línea con la concepción relacional de la experiencia por la que abogan estas autoras para comprender las implicaciones de la implementación de la eutanasia, hemos podido constatar que materializar ese regalo, para las solicitantes, conlleva transformaciones y costos para las y los profesionales^37^^,^^41^. Concretamente, implementar la ley (y sus tiempos) se hace, a menudo, sin disponer del tiempo profesional necesario, llegando incluso a desincentivar la participación de médicas y enfermeras^42^. Además, las y los profesionales, especialmente las enfermeras, cargan con el trabajo relacional de construir la confianza con la persona solicitante y, a la vez, acompañar a otras profesionales y, sobre todo, a la familia^32^. No obstante, a menudo se comparten estos nuevos espacios de reflexión y deliberación con estas familias sin disponer de los recursos profesionales, psicosociales y temporales para manejarlos^43^.

También, hemos puesto de manifiesto cómo la implementación de la LORE, a la vez que se ve afectada, afecta (“*invade y asalta*”, en palabras de nuestras participantes) incluso los tiempos bio-farmacológicos, generando tiempos cronológicos de una rapidez insólita, que no siempre van acompasados con los tiempos subjetivos^44^. Al mismo tiempo, esta ley resignifica no solo la muerte, sino también una práctica profesional que, en el contexto español, ha esperado y luchado mucho tiempo para dejar de contemplar el sufrimiento con impotencia^3^. 

Llegados a este punto, para profundizar en las implicaciones de los hallazgos aquí presentados, resulta relevante levantar la mirada de su contexto para atender a aquellos lugares en los que la regulación de la ayuda médica a morir es todavía un proyecto o un debate, como son los casos del Reino Unido o Argentina^45^. En el caso concreto de Latinoamérica, si bien la ayuda médica a morir se ha regularizado recientemente en países como Colombia, Cuba y Ecuador, lo cierto es que aún existe poca evidencia sobre su implementación práctica en estos contextos, y está muy centrada en aspectos legales^46^. En muchos otros países, la eutanasia es aún un hecho controvertido y altamente polarizado. A menudo, esta polarización se ubica a nivel moral, confrontando valores como la autonomía del paciente con la del profesional^47^. Sin embargo, el presente artículo permite precisamente anticipar también tensiones prácticas, que tienen que ver con la necesidad de un acompañamiento al cambio en el rol profesional de medicina y enfermería, mayoritariamente, mediante acciones como la formación en farmacología asociada a la ayuda médica a morir y al apoyo a la familia durante el impasse entre la solicitud, su aprobación y la prestación en sí misma.

En definitiva, en el presente trabajo hemos podido constatar cómo en una misma duración conviven e interactúan, con mayor o menor fortuna, múltiples tiempos: rápidos y lentos a la vez. En este sentido, en nuestros datos hemos visto cómo a menudo la ley trata de conjugar la multiplicidad de tiempos sobre un plano lineal, midiéndolos y recortándolos. Asimismo, Bal^23^ ha mostrado cómo la heterocronía puede dar lugar a un mayor sufrimiento, contradicción y parálisis. Sin embargo, esta misma autora también contempla que, si se consiguen entretejer las distintas temporalidades, la experiencia puede ser placentera y significativa^23^.

Por un lado, para comprender por qué las y los profesionales se sienten presionados^48^y se refieren a los tiempos objetivos de la ley, a menudo, como imposición, resulta pertinente retomar otro sentido del concepto heterocronía que, hasta donde los conocemos, Bal no recoge en sus escritos. Acuñado originariamente a finales del siglo XIX por el zoólogo Ernst Haeckel, este concepto se ha empleado de forma genérica en la biología del desarrollo para describir cambios, excepciones o mutaciones de naturaleza temporal. Es decir, se trata de un término que emerge de la comparación de dos temporalidades: una más conocida (o “basal”), referida al tiempo “normal” de evolución; y, otra, que es etiquetada como heterocrónica, a partir de su comparación con la primera, dado que esta última evolución sucede más rápido, más lento o durante más o menos tiempo que la original. 

Por ejemplo, el desarrollo del cerebro humano o del cuello de la jirafa se consideran heterocrónicos en relación con el de sus ancestros en tanto que son procesos que tardan más tiempo en terminar, aportando, en estos casos, ventajas evolutivas. De forma análoga, se podría considerar que la Ley Orgánica de Regulación de la Eutanasia es heterocrónica en tanto que altera los ritmos que hasta ahora se consideraban “normales” en el morir. Visto desde la multiplicidad de perspectivas que recogen nuestros datos, algunos tiempos son padecidos como heterocronías impuestas cuando no se sincronizan con otros tiempos del conjunto o ensamblaje de actantes implicadas en el proceso.

Desde un paradigma jurídico^49^, la ley impone un tiempo en lugar de componer los tiempos. Por lo tanto, resulta coherente que emerjan resistencias que podríamos llamar “temporales” a esta forma de constituir una nueva normalidad a través de la trama del tiempo objetivo. Por ejemplo, nuestras participantes hacen referencia a casos de colegas profesionales que, más o menos conscientemente, ejercen dilaciones para ganar tiempo social o personal, a veces en detrimento de la paciencia de los pacientes en su transición hacia una muerte digna.

Sin embargo, nuestros datos también reflejan instancias de composición. Nos referimos a aquellos relatos que dibujan equipos asistenciales capaces de conjugar todas las temporalidades presentes en el proceso eutanásico. Consecuentemente, deviene factible una transición armoniosa, en la que las distintas notas y ritmos se funden en una melodía, como diría Bergson^20^. Como ya nos adelantó la primera persona a quien entrevistamos, no debemos olvidar que “*de hecho, morirse es una canción*” (EE01:17, médica de hospital), en un sentido cuantitativo (lo que dura una canción), pero también cualitativo (cuando se armonizan los distintos ritmos).

En definitiva, tal y como apunta Foucault^50^, las heterotopías y, por extensión, las heterocronías pueden ser prisiones, pero también jardines. De hecho, nuestros datos nos invitan a pensar en la implementación de la eutanasia como heterocronía en un tercer sentido, que podemos llamar foucaultiano. Es decir, la Ley Orgánica de Regulación de la Eutanasia abre espacios y tiempos nuevos pero reales (a diferencia de las utopías). Los significados de estos “tiempos otros” están aún en construcción, ya que aúnan temporalidades diversas y que en las lógicas dominantes son excluyentes (como, por ejemplo: acompañar e incluso mejorar el bienestar de una persona a la vez que se adelanta su muerte). Finalmente, estos espacios temporales en construcción tienen la función de materializar la posibilidad de una muerte digna y, a la vez, denunciar que este no es el caso en la mayoría de los fallecimientos.

En resumen, la perspectiva de las personas profesionales implicadas en el proceso eutanásico nos lo presenta como una dinámica heterocrónica en, al menos, tres sentidos. En primer lugar, la prestación de la ayuda a morir es un proceso en el que coexisten múltiples temporalidades. No obstante, en segundo lugar, algunas de estas temporalidades pueden percibirse como heterocrónicas, en tanto que impuestas desde el exterior. Finalmente, en tercer lugar, la implementación de la eutanasia abre la posibilidad de que estas múltiples temporalidades sean ensambladas y compuestas en armonía en forma de una muerte digna, alrededor del deseo de anticipar la muerte y apropiarse del tiempo que queda^16^. 

## Epílogo

Mientras escribimos este artículo, leemos sobre la muerte de Ramón Bayés, en dos tiempos. Primero, las noticias tienden al homenaje, a ubicar la relevancia de su pensamiento para la psicología en general y, concretamente, para los cuidados paliativos. Unos días después, la prensa nos descubre que, además de ser un autor destacado y obsesionado con el buen morir, en sus últimos meses devino solicitante de eutanasia. 

Sin embargo, los artículos subsiguientes ya no hablan de él o de su obra, sino de sus últimos tiempos, vividos desde esta nueva subjetividad. Y nos hablan también de dolor, la vida y la muerte a través de numerosas referencias temporales: del terror a vivir demasiado, de los años de reflexión previa a la decisión, de la emergencia de la ley en un momento histórico en el que puede ser una herramienta para la prevención del suicidio, de la práctica de la dilación como objeción encubierta, de la falta de tiempo contemplado para la eutanasia en atención primaria, la falta de planificación que facilite la inyección del fármaco, de la impuntualidad el día de la prestación, y de los tiempos de espera^18^, de los tres largos meses de espera.

¿Qué hubiera hecho el Dr. Bayés de su propia experiencia? Lamentablemente, es una incógnita. Y no está de más que quede así, como un acontecimiento que es una heterotopía en tanto que denuncia, como fue toda su carrera, el largo camino que queda por recorrer hacia la muerte digna. Quizás sea mejor que su vivencia del morir resuene como una pregunta que impulse el aprendizaje y el viaje de profesionales, gestores, familias, etc., hacia el abordaje de la situación en toda su complejidad. Tal y como expone una de las personas que estuvo al lado del Dr. Bayés:

“La eutanasia no puede ser un acto con unos plazos que haya que cumplir. Es un acompañamiento, es un cuidar a la otra persona desde donde está emocionalmente”^51^.
