# Supplementary material for: “Todo fue muy rápido y muy lento, a la vez”: experiencias heterocrónicas de profesionales de la salud en la prestación de ayuda a morir en España
Source: Salud Colect. 2026 May 20;22:e5972. [Article in Spanish] doi: 10.18294/sc.2026.5972 (PMC13476420; doi:10.18294/sc.2026.5972)
Supplement: Translation

# Translation: “Everything was both very fast and very slow at the same time”: heterochronic experiences of healthcare professionals in the provision of aid in dying in Spain

**Authors:** Eduard Moreno-Gabriel, Maria Verdaguer, Patricia Beroiz-Groh, Xavier Busquet-Duran, Antonia Arreciado Marañón, Maria Feijoo-Cid, Miquel Domènech, Lupicinio Íñiguez-Rueda, Pere Toran-Monserrat

**Affiliations:** 1 Doctor en Psicología Social. Investigador, Unidad de Apoyo a la Investigación Metropolitana Norte, Fundación Instituto Universitario de Investigación en Atención Primaria de Salud Jordi Gol i Gurina, Mataró, España. emoreno@idiapjgol.info; 2 Doctora en Psicología Social. Investigadora, Unidad de Apoyo a la Investigación Metropolitana Norte, Fundación Instituto Universitario de Investigación en Atención Primaria de Salud Jordi Goli i Gurina, Mataró, España. mverdaguer@idiapjgol.org; 3 Doctora en Medicina. Médica, Hospital Germans Trias i Pujol, Badalona, España. pberoiz.germanstrias@gencat.cat; 4 Doctor en Medicina. Investigador, Unidad de Apoyo a la Investigación Metropolitana Norte, Fundación Instituto Universitario de Investigación en Atención Primaria de Salud Jordi Gol i Gurina, Mataró, España. Profesor, Universidad de Vic- Universidad Central de Cataluña, Manresa, España. xbusquet@umanresa.cat; 5 Doctora en Ciencias de la Enfermería. Profesora, Departamento de Enfermería, Universitat Autònoma de Barcelona, Barcelona, España. antonia.arreciado@uab.cat; 6 Doctora en Antropología Médica. Profesora, Departamento de Enfermería, Universitat Autònoma de Barcelona, Barcelona, España. maria.feijoo@uab.cat; 7 Doctor en Filosofía y Letras. Profesor, Departamento de Psicología Social, Universitat Autònoma de Barcelona, Barcelona, España. miquel.domenech@uab.es; 8 Doctor en Filosofía y Letras. Catedrático, Departamento de Psicología Social, Universitat Autònoma de Barcelona, Barcelona, España. lupicinio.iniguez@uab.cat; 9 Doctor en Medicina e Investigación Traslacional. Coordinador, Unidad de Apoyo a la Investigación Metropolitana Norte, Fundación Instituto Universitario de Investigación en Atención Primaria de Salud Jordi Gol i Gurina, Mataró, España. Profesor, Universitat de Girona, Girona, España. ptoran.bnm.ics@gencat.cat

**Keywords:** Euthanasia, Time Perception, Professional Practice, Spain

## Abstract

Since its enactment, Organic Law 3/2021 on the Regulation of Euthanasia has altered the experience and professional practice of numerous individuals within the healthcare field in Spain. This impact is evident in how these professionals perceive the various temporalities involved in the process. Using the notion of heterochrony, this article captures this multi-temporality through an interpretive phenomenological analysis of 29 interviews and three focus groups with professionals involved in medical aid in dying. The professionals report a multiplicity of overlapping and interacting times: the time of the law, the patient’s time, family time, bio-pharmacological times, and socio-historical time. Tensions are highlighted between the deadlines set by the law and the times experienced by applicants, or between bio-pharmacological temporalities and prevailing professional logics. The article also assesses temporal spaces to support families and transition toward an emerging professional paradigm. Managing this heterochrony poses a challenge for this new role. However, the assembly, synchronization, and composition of the different times could facilitate a harmonious implementation of the law, improving the experience of all involved.

## Introduction

“Death stands there in the background, but don’t be afraid. Hold the watch down with one hand, take the stem in two fingers, and rotate it smoothly. Now another installment of time opens, trees spread their leaves, boats run races, like a fan time continues filling with itself, and from that burgeon the air, the breezes of earth, the shadow of a woman, the sweet smell of bread. What did you expect, what more do you want? Quickly. strap it to your wrist, let it tick away in freedom, imitate it greedily. Fear will rust all the rubies, everything that could happen to it and was forgotten is about to corrode the watch’s veins, cankering the cold blood and its tiny rubies. And death is there in the background, we must run to arrive beforehand and understand it’s already unimportant.” *Julio Cortázar*^1^

With the approval of Organic Law 3/2021 regulating euthanasia (LORE),^2^ Spain became the first Mediterranean country to decriminalize assisted dying. This new legal framework entails a significant social change that directly affects healthcare professionals, who have consequently been called upon to reconsider their clinical practice.^3^^,^^4^

To account for the tensions experienced by professionals, studies on euthanasia frequently draw, in one way or another, on its etymology as a means of understanding them.^5^ In doing so, these studies locate professional dilemmas and conflicts in the relationship between “good death” and professional duty, contributing either to moral reflection or to discussions concerning the influence of legal factors on professional practice.^6^

However, the entry into force of the LORE and its implementation have introduced a new dimension to euthanasia in Spain, as it is gradually ceasing to be merely an abstract entity or a more or less tolerable possibility and is becoming a concrete clinical practice. In this regard, understanding what euthanasia means for professionals in Spain requires complementing studies concerned with how euthanasia is discursively constructed with praxiographic approaches capable of accounting for the practices that enact assisted dying in Spain.^7^ From this perspective, biomedical phenomena and objects such as euthanasia are no longer conceived as abstract and static entities toward which one may simply be for or against. Rather, euthanasia comes to be understood as a sociotechnical process in which multiple rhythms and practices are coordinated, and which are, in turn, experienced firsthand by the professionals directly involved in its implementation.^8^

As we will show below, practising euthanasia fundamentally involves managing and articulating temporalities in tension. Indeed, assisted dying may be understood as a translation into the end-of-life domain of particular forms of temporal orientation toward anticipation that characterize contemporary Western societies.^9^ More specifically, it entails materializing the so-called “desire to hasten death”, which emerges as a possibility within these sociohistorical contexts for situations marked by severe, chronic, and disabling suffering.^2^^,^^10^ In other words, from the praxiographic perspective adopted in this study, life and death are constructs that emerge within specific discursive contexts. At the same time, emphasis is placed on how these phenomena are the outcome of sociomaterial practices that, much like winding a watch, open up multiple temporalities which, when coordinated, make it possible to “run and arrive first” at death, in the words of the Argentine writer Julio Cortázar.

Thus, in the present study, we set out, in a sense, to unfold the fan of time and describe the different temporal experiences that, from the perspective of healthcare professionals, converge, are managed, and sometimes collide in the implementation of assisted dying since the entry into force of the LORE.^2^

### Contextual framework: time as a practical problem in the provision of assisted dying

Within the first few minutes of reading the legal text, it becomes evident that the identification, delimitation, and structuring of time constitute some of its primary objectives. On the one hand, eligibility for accessing this new right is largely defined by criteria such as the “chronic” nature of the condition or the presence of a “limited life expectancy”. On the other hand, in order to guarantee elements such as voluntariness, the consistency and deliberative nature of the decision, the right to appeal, and related safeguards, etc, the law repeatedly relies on the establishment of specific timeframes (for example, requiring two requests to be submitted at least 15 calendar days apart). Figure 1 provides a summary of the process, including the legally established time periods.

**Figure 1:** Process and timeline from the request for medical assistance in dying to its provision in Spain, according to the Organic Law Regulating Euthanasia.

Source: Own elaboration based on Altisent *et al*.^11^

Despite the legislative effort to objectify and clearly define the temporal dimensions of the assisted dying process, the first report produced by the Spanish Ministry of Health on the implementation of the LORE already identified time management and its consequences as one of the main challenges: “In some cases, the time elapsed between the second request and the consultant physician’s report was excessive”.^12^ Such delays continue to be documented in the most recent Catalan report, which indicates that one-third of applicants die before receiving the intervention, thereby increasing the distress experienced by those requesting assisted dying.^13^

This problematization of time is also present in the scientific literature, albeit with some variations and nuances.^14^ In an article published in 2021, Altisent *et al*.^11^ criticize the LORE precisely on temporal grounds. Reflecting a position that is relatively widespread among palliative care professionals, these authors question whether the “assistance in dying” addressed by the LORE could instead be limited to symptom management during the dying process, without entailing the hastening of death. Furthermore, their work suggests that lengthy waiting times for access to social benefits may influence the decision to request euthanasia. Finally, they identify both professional training and the availability of sufficient time for healthcare professionals to engage in the necessary deliberative processes as unresolved issues within the LORE.

In relation to the various Australian laws regulating euthanasia and voluntary assisted dying, Wagner^15^ goes beyond critiques focused on compliance with, or management of, temporal requirements to question the usefulness of adopting a linear conception of time as a framework for understanding the dynamics of acceptance and rejection surrounding such legislation and, by extension, euthanasia practice itself. Drawing on Deleuze and Guattari’s notion of the perpetual pendulum,^16^ Wagner proposes a non-linear and fluid understanding of temporality. From this perspective, she argues that the controversial nature of these laws can be understood as a dynamic process characterized by continual oscillation between acceptance and rejection. Such a framework moves beyond polarized positions, creating space for ethical debate as well as the ongoing discussion and evolution of legislation.

From this brief review of the legal, institutional, and academic texts that govern and reflect upon euthanasia, it becomes apparent that both the structuring of time and the very ways in which temporality is conceived are central to making the practice of medical assistance in dying possible.^17^ For this reason, the present article explores time from the perspective of the professionals involved in this practice, drawing upon a conceptualization of time that allows us to attend both to its multiplication and to its coordination within the singular context of assisted dying.^7^

### Conceptual framework: time as duration and heterochrony

As noted above, the LORE itself, as well as the broader body of texts concerning the provision of medical assistance in dying, incorporates and seeks to articulate two conceptions of time that are fundamental to understanding the dynamics of health, illness, and death: objective time and subjective time.

The Spanish psychologist Ramón Bayés, who recently underwent euthanasia and remains one of the leading theoretical references in the study of emotional aspects of palliative care, drew on this dichotomy from the work of William James to highlight the negative impact of waiting times on people with cancer and, more broadly, on almost all patients. Specifically, Bayés writes:

“William James already distinguished between objective time and subjective time. Although chronological time has been indispensable for developing the complex organization of the contemporary world-including the healthcare system-and for achieving extraordinary technological advances, for the individual, the time that truly matters in personal life is subjective time, whose duration is perceived as highly variable depending on one’s personal biography, expectations, and the events to which one is exposed at any given moment.”^18^

Applied to the case of euthanasia, although the LORE devotes considerable attention to establishing objective temporal frameworks through which the right to assistance in dying is assembled, for those involved in the process-and paraphrasing the famous quotation often attributed to Albert Einstein-the time that counts is precisely that which cannot be counted. Indeed, if euthanasia is to be understood as a practice or process (clinical, professional, and otherwise) rather than solely as a legal construct, objective time becomes of limited relevance.

To attend to this more subjective and dynamic dimension of temporality, it is useful to position ourselves against the predominantly quantitative conception of time associated with Einstein. At the opposite pole of the debate, though with the intention of complementing rather than refuting Einstein’s theories of time, Henri Bergson developed an alternative approach in the early twentieth century.^19^ More specifically, Bergson introduced the concept of *duration* (*durée*) to explain how time is experienced as something dynamic and continuous, fundamentally indivisible except through the intervention of an external perspective or device, such as a clock. Through the notion of duration, Bergson comes closer to capturing what is commonly referred to as “subjective time,” in which what matters is the lived experience of succession and the blending or transition between moments that gradually become indistinguishable from one another.^20^

Thus, when we approach the study of subjective time, we encounter a multiplicity of durations that may overlap, even though the doctrine of objective time is unable to conceive this of them as such.^21^ In the case of euthanasia as a healthcare practice, professionals often register, partition, and describe its temporalities through seemingly contradictory expressions, such as the one that gives this article its title. Consequently, understanding professional experiences and practices surrounding the provision of medical assistance in dying requires that we conceive of time as duration-as it is lived and felt, while encompassing other durations.^22^

The cultural theorist and artist Mieke Bal^22^identifies this multiplicity of time as a particularly distinctive feature of the present historical moment in Western societies. To describe this experience of multiple temporalities, she introduces the concept of *heterochrony*:

“This is how we come to realise that time is not homogeneous in our experience. Because we are so frequently multi-tasking, we are also multitemporalising: simultaneously living different paces in the of clock time. The concept of heterochrony helps account for the experiential differences facing, or being ruled by, clock time entail.”^23^

Bal first introduced the concept of heterochrony in the context of her exhibition *2MOVE: Migratory Aesthetics*, an effort to operationalize concepts that, like heterochrony, facilitate the kind of interdisciplinary work in the humanities pursued in the present study.^24^ For Bal, migration is the condition that characterizes our contemporary world, in which we are all constantly in motion, moving between haste, waiting, and stagnation, while navigating an unsettling present that is no longer grounded in a predictable future.^24^

Like the migrant, the professional involved in the provision of medical assistance in dying occupies a liminal position,^25^ situated amidst an overlap of temporalities and rhythms that may at times become paralysing. According to Greco and Stenner,^26^ this crossroads may also signal a broader shift in the prevailing understanding of the healthcare professional’s role (particularly that of physicians), from that of a “full-time life-saver” to that of a “companion in processes” capable of articulating multiple temporalities. Elsewhere, Stenner^27^argues that these new selves emerge from the capacity to position oneself between different perspectives, thereby facilitating the harmonization of the distinct temporalities each perspective brings. Following George H. Mead, this author reminds us that “we must be others if we are to be ourselves”,^28^ inhabiting what he terms a liminal sociality of transition. It is precisely this capacity to assume different roles and tempos that enables professionals, in this case, to participate in and bring into being collective processes such as euthanasia.

## Methods

The present study seeks to capture this diversity of ways in which time is (ac)counted for (now in a narrative sense) as it is experienced within the provision of medical assistance in dying in Catalonia (Spain). More specifically, we present this diversity from the perspective of the professionals involved in the assisted dying process.

These experiences were collected as part of a study funded by the Catalan Department of Health, approved by the Research Ethics Committee of the Jordi Gol Institute for Primary Care Research (reference code 22/094-P), and conducted during the first years of implementation of the LORE in Catalonia (2022-2024). As described in the published study protocol and descriptive article^29^^,^^30^, the core of the study consisted of 21 phenomenologically oriented individual interviews with healthcare professionals who had been directly or indirectly involved in at least one process of medical assistance in dying in Spain. Altogether, participants reported having been directly involved in 45 assisted dying procedures since the entry into force of the LORE. The sample included five nurses, ten physicians, three social workers, and three professionals from other occupational categories, all of whom participated in in-depth interviews.

The interviews lasted approximately one hour on average and were conducted face-to-face in a variety of locations, most commonly in private offices at the interviewees’ workplaces. Participants were allowed to choose the time and location that would enable them to share their experiences in a safe and confidential environment while also facilitating audio recording. All interviews were conducted, following the provision of informed consent, by a psychologist, with the support of a family physician. Given the family physician’s direct involvement as a key figure in the implementation of the LORE within primary healthcare services in the northern metropolitan area of Barcelona, the principal investigator acted as a non-participant observer during the interviews, taking field notes that informed subsequent analyses. At the same time, his professional position facilitated participant recruitment through a snowball sampling strategy guided by theoretical sampling, aimed at capturing the broadest possible range of professional profiles involved in the implementation of the law.

To capture participants’ social and psychological worlds, in-depth semi-structured interviews were employed. These were guided by an interview schedule developed from a literature review and a first round of eight exploratory interviews with key informants who possessed a broad overview of the law within the Catalan context, most of whom were members of the regional Guarantee and Evaluation Commission. However, the interview schedule was not used prescriptively; rather, conversations were conducted in a manner that maximized participants’ ability to tell their own stories, in accordance with the principles of Interpretative Phenomenological Analysis (IPA).^31^

Finally, given that the broader funded study envisaged the development of a good practice guideline, the interviews were complemented by six focus groups conducted across two phases. During the first phase, carried out concurrently with the phenomenological interviews, the primary objective was methodological triangulation. In the second phase, conducted after the completion of the phenomenological interviews, priority was given to the co-creation of recommendations and good practices for the implementation of the LORE. All interviews and focus groups were audio-recorded and transcribed verbatim by members of the research team and an external transcriber, with confidentiality safeguarded throughout the process.

As a result of the thematic analysis of the overall dataset, two articles have already been published in addition to the analysis presented here. First, in Verdaguer *et al*.,^30^ we identified the main sources of distress emerging from participants’ accounts, which served as the basis for the development of a good practice guideline currently under construction. Second, in Feijoo-Cid *et al*.,^32^ we highlighted the role of nursing professionals throughout the assisted dying process, as this professional group remains underrepresented and insufficiently recognized in both the academic literature and legal and official documents.

The present article draws on the data generated from the eight exploratory interviews, the twenty-one phenomenological interviews and the three focus groups conducted during the first phase of the study. These materials were analysed following the procedures of Interpretative Phenomenological Analysis (IPA),^33^^,^^34^ with the aim of exploring and understanding professionals’ lived experiences in depth. The analysis proceeded in a cyclical manner, attending both to themes emerging from the data and to the continuous comparison of these emergent constructs with the dataset as a whole. More specifically, the analysis began with an idiographic examination of each interview before moving toward a cross-case integration of findings. Initial impressions-generated both through listening to the interviews and through repeated readings of individual transcripts-revealed the centrality of lived time as one of the main categories (or thematic clusters) through which participants organized their narratives. Consequently, this overarching category and its different manifestations were used to structure the themes presented in the results section (i.e., the different temporalities), serving as analytical reference points against which the entire corpus was revisited and recoded. Four of the article’s authors participated in this analytical process, thereby facilitating data triangulation and enhancing the rigour of the analysis.

## Results

As is often the case in qualitative research, the first analytical intuitions began to emerge during the interviews themselves. When describing their experiences, participants frequently drew on direct or indirect references to their perception of the passage of time (or duration), as well as to the impact of other temporalities or rhythms that they tended to perceive as external to themselves. Beyond the detailed descriptions of their subjective experiences, it is noteworthy that participants explicitly referred to the co-existence of these different temporalities within a single moment:

“*Everything was both very fast and very slow at the same time”* [...] *Of course, if you look at the time between when we arrived at the hospital until these two people died, I reckon about two hours passed. But when it came to administering the injections, it all happened very quickly. We did not really know... I had some knowledge of the effects of these drugs, but not at these doses. They were extraordinary doses. But, of course, what you are trying to achieve is precisely that these people die, and that they die quickly. It is not sedation, it is not a process that unfolds over time... No. It is immediate. From the moment you administer it until... I think about ten minutes passed. We did not actually time it, but I would say it was around ten minutes from the moment we administered it... Yet it is true that the feeling I had, especially from the moment I was preparing the medication until the moment I entered the room and administered it, was like a... temporal distortion, you know?*” (EF:118, primary care nurse)

For this reason, we present below the different temporalities that emerged from our interviews, while seeking to keep their overlap visible. To this end, we invite the reader to imagine time as a spiral (Figure 2). At its centre lies the temporality of the patient moving toward death through their position as an applicant for assisted dying. This temporality is embedded within and traversed by other rhythms and temporal circles, ranging from the historical processes that enabled the emergence of the LORE to the temporalities required for the drugs used in the procedure to take effect.^35^

**Figure 2:** Temporalities in the implementation of medical assistance in dying.

Source: Own elaboration.

We begin our presentation of these temporalities with professional experience, as this constitutes the first-person perspective narrated by the study participants. We then follow the connections that professionals themselves establish in their accounts, linking their experiences to other temporalities and speeds in the following order: the patient-applicant, the family, bio-pharmacology, and historicity.

### Professional time: Intangible and extraordinary time

Beyond the end-of-life context, time frequently emerges as a key element in healthcare professionals’ accounts of their work experiences. Particularly in settings characterized by high clinical workloads, such as primary care in Spain, professional time is often perceived as a scarce resource. This scarcity gives rise to increasingly accelerated rhythms of work, accompanied by recurring narratives of distress among healthcare professionals. In our data, as in previous studies, temporality often appears in relation to a quantitative demand for more time:

“*And then there is an issue that, for me, is essential: time. The time that both the attending physician and the consulting physician have to dedicate when a request for euthanasia is made is time that they often simply do not have, and this generates a great deal of stress.* [...] *The insistence on pressing them to meet deadlines is mainly intended to ensure that everything is properly formalized, but the truth is that, as a system and as a model of care, we should organize things so that when a professional receives one of these requests, they actually have the time to deal with it. In other words, they should be able to clear their schedule for however many days they need to complete the process, because of the psychological burden it may entail... It requires time. I think that sometimes requests are denied simply because there is not enough time, and people end up saying: ‘Rejected.*’” (EE03:88, legal expert, woman, member of the Guarantee and Evaluation Commission)

As reflected in this participant’s account, the lack of sufficient time is identified as a possible explanation for both explicit and implicit refusals by non-objecting professionals to participate in the implementation of medical assistance in dying. In turn, these refusals or delays contribute to the temporal extension of the process itself. However, participants’ accounts move beyond a merely quantitative assessment of the time required to carry out the procedure. Instead, they emphasize the intensity of the task and the ways in which it exceeds the temporal boundaries formally allocated to it, extending into both the past and the aftermath of the intervention:

“*The first one*, [name of applicant], *I spent fifteen days unable to sleep. I kept thinking: ‘I cannot afford to miss anything.*’” (EE01, hospital physician)

“*And then, above all, time. Another very serious issue, I think, is that... You know what is happening with the current workload in healthcare... It is very difficult to devote the time needed to process, reflect on, and accompany what is happening when you are under so much clinical pressure. Sometimes colleagues have had to carry out a MAiD procedure and then immediately go on a home visit. That is a lot. You need to stop and metabolize the experience of that accompaniment, that beautiful or emotionally moving image you have witnessed; you need time to absorb it. And sometimes they simply do not have that time, especially in primary care. I know this because many of my friends work in primary care and face precisely this difficulty: enormous workload pressures, a frantic pace, and yet a genuine desire to accompany patients and respond authentically to their needs. But I wonder whether they are given enough space. Perhaps one form of care would be to ensure that someone who performs a MAiD procedure does only that on that particular day. Perhaps it means allowing enough time afterwards so that you can go for a walk on the beach or sit down for a cup of tea. Or a glass of wine, for that matter... But to stop. I have the feeling that, sometimes, that simply is not possible*.” (EE05:70, psychologist)

Although certain similarities with other clinical practices can be identified, participants described the provision of medical assistance in dying as a distinctly different undertaking-one that involves preparation, accompaniment, metabolization (to use the term employed by the participant cited above), and even a specific pause. From the perspective of the professionals interviewed, implementing euthanasia requires forms of time that are both qualitatively and quantitatively distinct and, at the same time, difficult to accommodate within the current organization of healthcare practice in Spain. Beyond being “intangible,” these temporalities are perceived as unusual, falling outside the logic and planning of contemporary healthcare systems:

“*Of course, I have been involved in 24* [euthanasia procedures] *in one way or another. That means many hours, many hours, many weekends, public holidays, extending beyond vacation periods because there is an ongoing case.* [...] *But then my colleagues have needs, and sometimes those needs are rather unusual. And what happens? You log on, you talk, of course... All of this is intangible, but it is there. And, honestly, it represents a huge amount of work for me*.” (EE01:10, hospital physician)

In this account, the participant implicitly alludes to the effects and additional burden generated by the conscientious objections mentioned earlier. At present, in Catalonia, the number of professionals with extensive knowledge of the temporalities and procedural demands involved in euthanasia remains relatively limited. As a result, the implementation of the process inevitably depends on the time and availability of these individuals, who often act as reference professionals for colleagues seeking guidance throughout the procedure. Consequently, their time becomes stretched and saturated in ways that are simultaneously profound and largely invisible.

### The (im)patient’s time: toward the applicant’s un-waiting

As illustrated in the excerpts concerning the professional perspective, among the demands articulated by healthcare professionals regarding not only the quantity but also the quality of the time required for the provision of medical assistance in dying, the importance of an open-ended and relational process emerges-namely, accompaniment and the development of a trusting physician-patient relationship:

P1: “*There is also the issue of the relationship... it is linked to trust. Trust is not simply a matter of the patient trusting the physician; it is also about the physician trusting the patient. Trust is mutual, and that is very important*.”

P2: “*And it takes time to develop. You do not walk into a consultation and instantly have trust.*”

P1: “*And that makes everything much easier.*” (GD02:157, healthcare professionals participating in a focus group)

In this way, we can see how the perspective of the applicant enters into the duration of the professional providing care and requires reciprocity in order for the process to unfold more smoothly. Although it is difficult to quantify the time necessary to establish trust-since it depends on the interaction and coordination of at least two perspectives-professionals clearly recognize both its importance and its temporal dimension.

From the professional perspective, the temporality of applicants can be divided into at least two moments-or, more accurately, two positions, since they frequently overlap and coexist: time as a patient and time as an applicant. Given that our research focused on experiences during the implementation of the LORE, our data contain relatively few references to the period preceding the decision to apply for euthanasia. Nevertheless, participants’ accounts suggest a gradual process in which individuals move from the position of “patient” (in a double sense: as someone embedded within a healthcare system that treats them as such and, at the same time, as someone capable of patiently waiting and accepting the slow progression of illness) toward a more “active” subjectivity, in which they take the initiative and decide to request euthanasia. In doing so, the position of applicant is superimposed upon rather than replacing that of patient, giving rise to a person who becomes, in a sense, increasingly “impatient”-someone who can no longer wait.

“*For patients, once they have made up their minds, the waiting period feels long because everything is already very clear in their minds*.” (EF02:739, primary care nurse)

“*I remember that, in the early requests, when people submitted the second request, they often used the observations section to complain-and there were many complaints. They would write things like: ‘How much longer do I have to wait? I have already made my decision. Why are you making me wait so long?’ Because once you have taken that step, I imagine the waiting must feel endless for the person requesting the procedure*.” (GD01:61, legal expert, member of the Guarantee and Evaluation Commission, focus group participant)

As the observations referred to by this participant illustrate, the determination-and at times the urgency-experienced by applicants often clashes with the temporal requirements established by the law. However, as we will see below, there is another perspective from which these legally mandated waiting periods acquire a different meaning.

### Family time: between understanding and hope

“*I think it helps families to understand, because some families will understand and others will not. But I do think that this period helps them come to terms with their relative’s decision. I know that, for the family member [requesting euthanasia], it is a real burden-if I tell you that I have had enough, do you still make me wait another month? Damn. I understand it from the patient’s perspective, but I do think that this time helps the family.*” (EF01:66, hospital palliative care nurse)

Once again, this excerpt reveals a contrast and juxtaposition of perspectives, emotions, and effects associated with the same temporal interval. Specifically, the participant refers to the 15-day period established by law for applicants to reaffirm their decision. From the applicant’s perspective, this waiting period may be experienced not only as unnecessary but even as an additional burden. However, according to the professionals interviewed, this same period is experienced differently by family members, insofar as it creates a space in which they can come to understand their relative’s decision.

Within this interplay of interacting temporal perspectives-the law, the patient, the family, and others-forms of synchronization may also emerge that temporarily displace the applicant from the centre of the process, despite the fact that, on paper, they are the person meant to guide it. For example, these legally mandated waiting periods also allow time for professional reflection (perhaps compensating for the temporal scarcity described earlier) and, more or less consciously, give rise to hopes shared by some professionals and family members:

“*The reflection period is not really for the patient, because they are already completely certain and have reflected on it more than enough. In fact, I used to say that I should stop calling it that. It is for the professional-to undertake the journey of deciding whether they can actually do this work and whether they can carry it through to the end*.” (EF01:91, hospital palliative care nurse)

“*When we learned the date...* [...] *You speak with the patient and you always think: ‘They will change their mind...’ But no. You let time pass... and still no.* [...] *And the family, too, waiting for them to say no at the last moment. The family was hoping*.” (EF22:30, primary care nurse)

### Bio-pharmacological time: “I don’t have 45 days”; “I have therapeutic reserves, a therapeutic arsenal”

Beyond personal, familial, and professional experiences, participants’ accounts invite us to adopt a symmetrical perspective on the euthanasia process and to understand it as a sociotechnical practice.^36^ More specifically, participants show how this praxis articulates the lived temporalities of those involved with the intervention of what, for analytical purposes, we may call “non-human” temporalities.

First, the perspective of applicants, as perceived and narrated by healthcare professionals, often incorporates the possibilities and temporal constraints imposed by bodily physiology (“I don’t have 45 days”).

“*There is no need to make someone wait 45 days while countless committees sign paperwork. This is another issue that generates a great deal of distress. ‘Why do I have to wait 45 days? It seems unfair to me, it’s not right, I don’t have 45 days...’ We are adding another layer of anguish because people do not know whether they will survive long enough to complete the process.*” (EF17:36, primary care nurse)

“*It was not until the end of October that we managed to submit the first official request... In the end, we did not get to... We completed the second request, but there was a premature death because the patient developed a multisystem infection. We transferred him home and I initiated palliative sedation.*” (GD03:40, primary care physician, focus group participant)

“*Everything was in place, but because of the timing, we did not make it. Over a single weekend-and it was not even during the working week, I was not there either-the patient had to be attended by professionals who did not know him very well. It happened from Saturday night into Sunday morning. He developed an upper gastrointestinal haemorrhage and entered a critical condition at home.*” (FG01:51, primary care physician, focus group participant)

As suggested by this last quotation, the temporalities of the body-in-illness and their possibilities also come into contact-and sometimes into conflict-with other temporal dynamics. Here, we see how the social organization of working time does not always succeed in synchronizing with the “timings” of, for example, an ageing and increasingly fragile digestive system. Clinical frailty may generate sudden imbalances that require urgent intervention during weekends and in the absence of the trusting relationship cultivated over months by the professional responsible for the case.

On the other hand, some bodies endure until the scheduled date of the procedure, and perhaps beyond. As illustrated in the following excerpt, a more strictly biomedical perspective draws upon material elements-such as the available “therapeutic arsenal”-to express disagreement with the person’s decision, precisely in relation to the amount of time that person ought to continue living (“there are still options available”):

“*On the contrary, the law is clarifying. What matters is the person’s will and the euthanasia context. It is much more complicated in relation to critical care patients, for example when dealing with treatment-limiting decisions. That is where we really struggle. ‘Because I’m not sure’, ‘I think there are still options available’, ‘I still have therapeutic reserves, a therapeutic arsenal that can be used’*.” (GD02:214, primary care nurse, focus group participant)

In those cases where the day of the procedure finally arrives, pharmacology assumes an even more prominent role, largely because of its speed. In many instances, the memory that lingers the longest and unexpectedly resurfaces over time is a moment marked by a peculiar sense of temporal unreality, whether due to the rapidity or the apparent slowness with which the drugs take effect.

“*It affected me more afterwards. At the time, I felt calm, but that night the image of him came back to me-the change in his colour. It is immediate. That was what really struck me: how quickly death arrives.*” (GD01:39, primary care nurse, focus group participant)

“*From that point on, it is as I said before: time disappears, there is no measure of time anymore. You try to make things as easy as possible, doing what you believe needs to be done with the injection and the administration of the drugs, but you lose your sense of reality; it is a moment in which reality slips away. And if, on top of that, there is even the slightest complication-as when you administer the medication and think, ‘My goodness, he is still saying goodbye’-you start to panic.* [...] *Afterwards, everything was straightforward, very straightforward. Straightforward, but as a physician you desperately want it to be over quickly because you are suffering. You place the stethoscope on the chest and it is still beating, the heart keeps beating. ‘Oh God, what have I done wrong?’ You start wondering whether you will have to open the second kit because one has not been enough. You think many minutes have already passed, and then you listen again: the heart is still beating. You panic, truly. For me, it was such a difficult moment. Then the reference professional says, ‘No, everything is fine, we just have to wait. Let’s follow the clock.’ But during what was actually two minutes, I thought at least ten had passed. And the heart kept beating. Because what you want is for it to be over; you want to leave, you want to leave. You have done everything you had to do, your heart is heavy, and you just want to leave, no matter how.*” (EF08:28, primary care physician and psychiatrist)

This excerpt is particularly revealing of the temporal distortion (“time disappears”) that accompanies the entire process and becomes especially evident at the moment of the procedure itself. At the same time, we can observe how this participant finds themselves positioned at the intersection of multiple temporalities, attempting to bring a degree of coherence to them: the professional temporality that, “as a physician,” demands swiftness; the technical-pharmacological temporality, which offers an expectation of order and predictability but is itself shaped by a heart that continues to follow its own tempo, its own rhythm, beyond the objective time measured by the clock; etc.

### Socio-historical time

The tension conveyed in the vivid account presented above can be explained, in part, by the collision of two temporalities that give rise to the perception of rapidity associated with the effects of euthanasia medications. This sense of acceleration emerges through comparison between the action of the drugs (or their dosages) used in euthanasia and the duration of procedures that, prior to the enactment of the law, constituted the professional norm, such as palliative sedation.

“*You have witnessed someone die like that, in no time at all. Because, of course, I see many sedation processes here, but those are very gradual; they are not this fast. And it all happens from this level of awareness of: ‘I am ready now, we can begin.’ That was what struck me. But by the following day, I was fine.*” (EF06:63, hospital spiritual care professional)

In other words, the impact of the temporalities dictated by the medications used in euthanasia emerges within a specific context that cultivates a particular “level of awareness,” one prepared to synchronize with certain rhythms of dying. By contrast, the relatively short period that elapsed between the implementation of the LORE and the interviews conducted for this study (between two and three years) has not allowed the professionals responsible for administering these medications sufficient time to receive training and acclimatize to these new pharmacological temporalities. Indeed, one of the most significant effects of the law concerns a transformation in professional subjectivities, which are currently navigating a transition toward a new clinical landscape:

“*People confuse the treatments. For example, in this case, another thing that happened was that the team had no doubts about what they had to do or how they had to do it, but the reality was that they had never done it before. It involved entirely new medications, and nobody had received any training on how to use them or on the kinds of situations that might arise.* [...] *It is not the same as administering palliative sedation, where you know you are giving Buscapina®, fentanyl, or midazolam and you know what effect they will have, as administering propofol or* [unintelligible], *which is something completely different.* [...] *The physicians had never before administered medication whose purpose was to bring about death*.” (EE05:36, psychologist)

At the same time, this “period of social maturation” (to paraphrase the participant quoted below), which is currently regarded as insufficient for the emergence of a fully trained professional community, has nevertheless been sufficient for the enactment of a particular law. Both the timelines (44 days) and the procedural sequence (prior authorization versus subsequent review) established by the law reflect what its architects considered tolerable within the prevailing socio-historical and sociotechnical context. As the following quotation illustrates, these arrangements were institutionalized through the Medical Assistance in Dying Software (*Programa de Prestació d’Ajuda per Morir*, PRAM). The same quotation also reinforces the socio-historical dimension of euthanasia by referring to developments in other countries (the Netherlands, Belgium, etc.), thereby opening the possibility that the Spanish context may evolve differently in the future.

“*The process itself is painful.* [...] *It is also painful because the law, in its implementation, is painful. The process takes at least 44 days if everything goes well. Then there are those who argue that we have a law based on prior authorization... The Dutch and Belgian laws are different, in the sense that oversight occurs afterwards. This generates suffering, not only because of all the bureaucracy-and in Catalonia this is particularly evident, because the PRAM software developed by the regional health authorities at the time is a disaster.* [...] *Beyond the procedure itself, which is cumbersome and lengthy, this makes everything even more difficult. And then there is what we discussed earlier regarding progression: some argue that this generates additional suffering because the physician-patient relationship is disrupted and invaded by an administrative process that should not exist. The truth is that the level of trust within society made this law possible. Personally, I would favour a different trajectory, but perhaps society was not sufficiently mature to allow it... Laws are always approximations, and this is the first approximation that has been achieved here, with all its limitations. It was simply not possible to enact a different law. That, too, generates suffering: the way the law is designed. The bureaucracy, the waiting times, prior authorization, subsequent review, having to go through a commission...*” (EE04:16, bioethicist psychologist, member of the Guarantee and Evaluation Commission)

Ultimately, participants’ accounts highlight not only the multiple temporalities that traverse the euthanasia process, but also the differences in the scale of those temporalities, ranging from the rhythm of a beating heart to the pace of social maturation, passing through the deadlines established by law. Yet, beyond this multiplicity of times, what stands out most is the way these temporalities interact with one another. In this regard, a recurrent theme across the interviews is the perception that bureaucratic temporalities “disrupt and invade” (to use the participant’s own words) the process of building a social bond such as the physician-patient relationship-a relationship constituted by subjectivities that are themselves in transition.

## Discussion

“Think of this: When they present you with a watch they are gifting you with a tiny flowering hell, a wreath of roses, a dungeon of air. They aren’t simply wishing the watch on you, and many more, and we hope it will last you, it’s a good brand, Swiss, seventeen rubies; they aren’t just giving you this minute stonecutter which will bind you by the wrist and walk along with you. They are giving you-they don’t know it, it’s terrible that they don’t know it-they are gifting you with a new, fragile, and precarious piece of yourself, something that’s yours but not a part of your body, that you have to strap to your body like your belt, like a tiny, furious bit of some­thing hanging onto your wrist. They gift you with the job of having to wind it every day, an obligation to wind it, so that it goes on being a watch; they gift you with the obsession of looking into jewelry-shop windows to check the exact time, check the radio announcer, check the telephone service. They give you the gift of fear, some­one will steal it from you, it’ll fall on the street and get broken. They give you the gift of your trademark and the assurance that it’s a trademark better than the others, they gift you with the impulse to compare your watch with other watches. They aren’t giving you a watch, you are the gift, they’re giving you yourself for the watch’s birthday.”^1^

In this study, we have gathered the perspectives of professionals involved in the implementation of the Organic Law Regulating Euthanasia in Spain, highlighting the importance they attribute to the perception of time in describing their experiences. Participants referred to and described bureaucratic, professional, familial, pharmacological, and social temporalities, as well as, of course, the temporality of the patient in their becoming an applicant for euthanasia.

With regard to the latter, professionals reported that applicants often described the possibility of anticipating their death as “the greatest gift you could have given me” (EF22:30, primary care nurse). Yet, as Cortázar warns us in the epigraph above, certain gifts-and a watch in particular-may constitute a “flowering hell,” insofar as they weave new sociomaterial relations that entail emerging obligations, responsibilities, and needs. In line with this observation, several studies examining professional experiences of implementing medical assistance in dying have characterized these experiences as marked by profound ambivalence and contradictory emotions.^37^^,^^38^

Throughout the interviews conducted as part of our project, we were able to capture some of these tensions, introduced in this case by the law and the temporalities it establishes, as well as the forms of distress associated with them.^30^ At the same time, however, professionals also reported receiving expressions of gratitude from patients for this “gift.” Indeed, the implementation of the law enables many individuals to move from the position of patient to that of applicant for euthanasia, generating a new form of waiting but also a sense of empowerment.^39^ In this regard, Pesut *et al*.^40^ explain the gratitude frequently expressed by applicants: setting a date and a timeframe for dying means, in a certain sense, regaining control over the time that remains and, by extension, over one’s own life.

In line with the relational conception of experience advocated by these authors for understanding the implications of implementing euthanasia, we found that materializing this gift for applicants entails transformations and costs for healthcare professionals^37^^,^^41^. More specifically, the implementation of the law (and the temporalities it establishes) is often carried out without the professional time required to do so adequately, to the extent that it may even discourage physicians and nurses from participating in the process.^42^ Moreover, healthcare professionals-particularly nurses-bear much of the relational work involved in building trust with applicants while simultaneously supporting other professionals and, above all, family members.^32^Yet these newly created spaces for reflection and deliberation are frequently shared with families without the professional, psychosocial, and temporal resources necessary to manage them effectively.^43^

We have also shown how the implementation of the LORE, while itself being affected by existing temporalities, simultaneously affects them, “invading” and “disrupting” them, in the words of our participants, even the bio-pharmacological temporalities. In doing so, it generates chronometric times of unusual rapidity that are not always synchronized with subjective experiences of time. At the same time, the law reconfigures not only the meaning of death but also a professional practice that, in the Spanish context, has long sought and struggled to move beyond witnessing suffering with a sense of powerlessness.^3^

At this point, in order to further explore the implications of these findings, it is useful to move beyond the Spanish context and consider settings where the regulation of medical assistance in dying remains either a political project or an ongoing public debate, such as the United Kingdom and Argentina.^45^ In Latin America specifically, although medical assistance in dying has recently been regulated in countries such as Colombia, Cuba, and Ecuador, empirical evidence regarding its practical implementation remains scarce and is largely focused on legal dimensions.^46^ In many other countries, euthanasia continues to be a highly controversial and polarized issue. Such polarization is often framed in moral terms, opposing values such as patient autonomy and professional autonomy.^47^ However, the present study allows us to anticipate practical tensions as well, particularly those associated with supporting changes in the professional roles of physicians and nurses. Such support may include, among other measures, training in the pharmacology of medical assistance in dying and in providing support to families during the interval between the request, its approval, and the procedure itself.

Ultimately, our findings reveal how multiple temporalities coexist and interact-sometimes successfully, sometimes not-within a single duration: fast and slow at the same time. In this regard, our data suggest that the law frequently attempts to reconcile this multiplicity of temporalities within a linear framework by measuring, segmenting, and delimiting them. Likewise, Bal^23^has shown how heterochrony can generate suffering, contradiction, and paralysis. Yet the same author also argues that, when different temporalities can be successfully interwoven, experience may become meaningful and even pleasurable.^23^

On the one hand, in order to understand why healthcare professionals often feel pressured^48^ and frequently describe the objective timelines established by the law as an imposition, it is useful to revisit another meaning of the concept of heterochrony-one that, to our knowledge, is not explicitly addressed in Bal’s work. Originally coined in the late nineteenth century by the zoologist Ernst Haeckel, the term has been widely used in developmental biology to describe temporal changes, deviations, or mutations in developmental processes. More specifically, heterochrony emerges through the comparison of two temporalities: one regarded as familiar or “baseline,” corresponding to the normal course of development, and another identified as heterochronic because it unfolds more quickly, more slowly, for a longer period, or for a shorter period than the former.

For example, the development of the human brain or the giraffe’s neck is considered heterochronic in relation to that of their ancestors because these processes take longer to complete, thereby conferring evolutionary advantages. By analogy, the Organic Law Regulating Euthanasia may itself be understood as heterochronic insofar as it alters the rhythms that have traditionally been regarded as “normal” in the process of dying. Viewed from the multiplicity of perspectives represented in our data, certain temporalities are experienced as imposed heterochronies when they fail to synchronize with the other temporalities that compose the assemblage of human and non-human actors involved in the process.

From a legal perspective,^49^the law imposes time rather than composing temporalities. It is therefore unsurprising that forms of resistance emerge which might be described as “temporal” resistance to this attempt to establish a new normality through the framework of objective time. For example, our participants referred to cases involving professional colleagues who, more or less consciously, introduced delays in order to gain social or personal time, sometimes at the expense of patients’ capacity to wait during their transition toward a dignified death.

However, our data also reflect instances of composition. We refer here to those accounts that portray healthcare teams capable of bringing together all the temporalities involved in the euthanasia process. Consequently, a harmonious transition becomes possible, one in which the different notes and rhythms merge into a melody, as Bergson would put it.^20^ As the very first person we interviewed anticipated, we should not forget that “in fact, dying is a song” (EE01:17, hospital physician), both in a quantitative sense (the duration of a song) and in a qualitative one (when different rhythms are brought into harmony).

Ultimately, as Foucault^50^ suggests, heterotopias and, by extension, heterochronies can be prisons, but they can also be gardens. Indeed, our data invite us to conceive of the implementation of euthanasia as a heterochrony in a third sense, which we might call Foucauldian. That is, the Organic Law Regulating Euthanasia opens up new yet real spaces and temporalities (unlike utopias). The meanings of these “other times” are still under construction, as they bring together diverse temporalities that are often treated as mutually exclusive within dominant frameworks (for example, accompanying and even enhancing a person’s well-being while simultaneously hastening their death). Finally, these emerging temporal spaces serve the function of materializing the possibility of a dignified death while also exposing the fact that such a possibility remains absent from most dying trajectories.

In summary, the perspectives of professionals involved in the euthanasia process present it as a heterochronic phenomenon in at least three senses. First, medical assistance in dying is a process in which multiple temporalities coexist. Second, some of these temporalities may be experienced as heterochronic insofar as they are perceived to be imposed from the outside. Third, the implementation of euthanasia opens up the possibility that these multiple temporalities may be assembled and composed harmoniously in the form of a dignified death, structured around the desire to anticipate death and reclaim ownership of the time that remains.^16^

## Epilogue

While writing this article, we learned of the death of Ramón Bayés, in two temporal moments. Initially, media reports focused on paying tribute to him and situating the significance of his contributions to psychology in general and, more specifically, to palliative care. A few days later, the press revealed that, in addition to being a prominent scholar deeply preoccupied with the question of dying well, in his final months he had become an applicant for euthanasia.

Subsequent articles, however, no longer focused on Bayés or his work, but rather on his final period of life, lived from this new subject position. They also spoke of pain, life, and death through numerous temporal references: the fear of living too long; the years of reflection preceding his decision; the emergence of the law at a historical moment in which it could serve as a tool for suicide prevention; delay as a form of covert conscientious objection; the lack of time allocated for euthanasia in primary care; the absence of planning to facilitate the administration of the medication; the lack of punctuality on the day of the procedure; and the waiting times^18^-three long months of waiting.

What would Dr. Bayés have made of his own experience? Unfortunately, this we will never know. Or, perhaps this is the best-case scenario as an event that constitutes a heterotopia insofar as it denounces-just as his entire career did-the long road that still remains toward achieving a dignified death. Perhaps it is better that his experience of dying continues to resonate as a question that encourages learning and reflection among professionals, policymakers, families, and others, prompting them to engage with the situation in all its complexity. As one of the people who accompanied Dr. Bayés expressed:

“Euthanasia cannot be an act governed by deadlines that simply have to be met. It is an accompaniment; it is caring for another person from where they are emotionally.”^51^
